# Inferring Centrality from Network Snapshots

**DOI:** 10.1038/srep40642

**Published:** 2017-01-18

**Authors:** Haibin Shao, Mehran Mesbahi, Dewei Li, Yugeng Xi

**Affiliations:** 1 Department of Automation, Shanghai Jiao Tong University and the Key Laboratory of System Control and Information Processing, Ministry of Education of China, Shanghai 200240, China; 2 Department of Aeronautics and Astronautics, University of Washington, Seattle, WA, 98195-2400, USA

## Abstract

The topology and dynamics of a complex network shape its functionality. However, the topologies of many large-scale networks are either unavailable or incomplete. Without the explicit knowledge of network topology, we show how the data generated from the network dynamics can be utilised to infer the tempo centrality, which is proposed to quantify the influence of nodes in a consensus network. We show that the tempo centrality can be used to construct an accurate estimate of both the propagation rate of influence exerted on consensus networks and the Kirchhoff index of the underlying graph. Moreover, the tempo centrality also encodes the disturbance rejection of nodes in a consensus network. Our findings provide an approach to infer the performance of a consensus network from its temporal data.

Centrality is designed to identify the most important nodes in a network and has been examined for several decades^1^,^2^,^3^. A node with a larger centrality value is considered more influential in a network^4^. The network topology plays a paramount role in centrality metrics, for instance, through its degree (the number of connections incident upon a node), eigenvector (based on the idea that connections to high-scored nodes contribute more to the score of the node than connections to low-scored nodes), betweenness (quantifies the number of times a node acts as a bridge along the shortest path between two other nodes), closeness (the reciprocal of the sum of its distances from all other nodes), and *k*-shell (or *k*-core of a graph is its maximal connected subgraph in which all nodes have degree at least *k*), etc. However, the topology of many large-scale networks could be unavailable, incomplete or unreliable. Even when the network topology is known, it might be expensive to compute its centrality index using global information. To this end, we examine whether we can compute the network centrality without the explicit knowledge of network topology and whether each individual node can infer its role from local observations. In addition to the network topology, the dynamics provides an alternative ingredient for identifying the role of nodes in a complex network. Recently, more links between centrality metrics and the network dynamics have been explored, such as in the context of percolation centrality^5^, control centrality^6^, and grounding centrality^7^, etc.

In this paper, we focus on consensus networks, a type of complex network whose components are diffusively coupled, and provide an elegant prototype for collective behaviors such as flocking of birds, schooling of fish, and phase transition in self-propelled agents^8^. We introduce a novel metric tempo centrality to quantify the influence of nodes in a consensus network, and show that the proposed metric can be computed from the temporal data of the network, even when the network topology is unavailable. An algorithm is subsequently given for the computation of tempo centrality. Moreover, the tempo centrality is shown to have a close correlation with both convergence rate and functional robustness of consensus networks.

## Results

### Tempo Centrality

Consider a networked system with 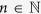 nodes depending on the application. Each agent 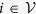 has a state at time *t* denoted by *x*_*i*_(*t*) that could represent position, temperature, or opinion. The interaction topology amongst agents is dictated by a graph 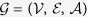 with a node set 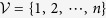, an edge set 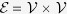, and an adjacency matrix 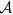, respectively. The adjacency matrix 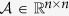 is such that 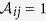 when 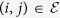 and 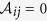 otherwise. The networks discussed in this paper are assumed to be simple, undirected and connected^9^.

We discuss the consensus dynamics


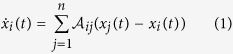


that has been extensively examined in distributed coordination of multi-agent systems^10^,^11^,^12^,^13^, opinion formation in social networks^14^,^15^ and synchronization amongst oscillators^16^. We shall refer to network adopting consensus dynamics (1) as the consensus network. We say consensus is achieved for a consensus network when 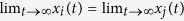 for all 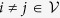
10. The consensus dynamics (1) provides an elegant prototype for the emergence of synchronization^17^.

Controlling a consensus network (steering the states of all the agents to a desired value) can be achieved by influencing a group of agents 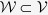 and the effect of control depends on the selection of the set 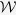. In this paper, we are interested in understanding the role of agents in a consensus network when acting as anchors through which the external inputs exert influence on the network.

Denote the set of external inputs as 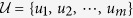, where 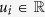 and *i* ∈ {1, 2, …, *m*}. Those agents who are directly influenced by the external inputs are referred to as the leader agents (or informed agents). The set of leader agents is denoted by 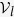. Denote by 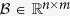 as the influence matrix such that 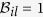 if and only if there exists 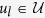 such that the agent 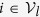 is directly influenced by *u*_*l*_, and 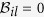 otherwise. It is assumed that each agent can be directly influenced by at most one external input. Thus the sum of each row of 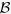 is less than 1 and there exists a one-to-one correspondence between 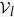 and 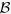. Denote the influence matrix determined by a node set 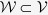 as 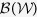. The individual behaviour is thus shifted to the following influenced consensus dynamics as a result of the external input,


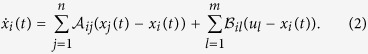


The network state, denoted by ***x***(*t*) = [*x*_1_(*t*), *x*_2_(*t*), …, *x*_*n*_(*t*)]^Τ^, characterises the behaviour of the overall system where ^Τ^ represents the transpose operation. The dynamics of the network state with external inputs is then


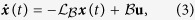


where ***u*** = [*u*_1_, *u*_2_, …, *u*_*m*_]^Τ^, 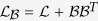, and 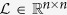 represents the graph Laplacian or Kirchhoff matrix satisfying 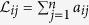 for all *i* = *j* and 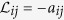 for all *i* ≠ *j*. The complex network with the influenced consensus dynamics (2) is referred to as the influenced consensus network.

An example of the structure of an influenced consensus network is shown in Supplementary Figure 1, where agent 1 is directly influenced by an external input 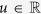 and therefore is a leader agent. In this example, the influence matrix is


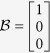


and the corresponding influenced consensus dynamics is


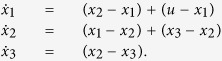


On the one hand, the evolution of the influenced consensus network (3) depends on the type of external inputs. The set of external inputs 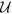 is homogeneous if *u*_*i*_ = *u*_*j*_ for all 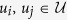 and heterogeneous otherwise. If the external inputs are homogeneous, then consensus is achieved on the value of the external input, namely, lim_*t*→∞_*x*(*t*) = *u*_*i*_**1** where 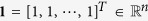 and *i* ∈ {1, 2, …, *m*}^11^. Clusters (where the states of agents converge to several distinct values) would emerge when external inputs are heterogeneous^18^. The results in this paper are available for both homogeneous and heterogeneous inputs. We do assume however that the external inputs are time-invariant which can be considered as the belief of a zealot^19^ or the opinion of stubborn individuals or political leaders^15^.

On the other hand, the dynamics of the influenced consensus network is also shaped by the influence matrix 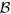. Note that 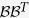 is a diagonal matrix with only *ii*-th entry 1 where 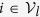, and 0 for other entries. The matrix 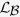 is then derived from the perturbation of the graph Laplacian by the diagonal matrix 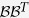; it is therefore referred to as the perturbed Laplacian matrix. The perturbed graph Laplacian of an undirected graph is symmetric; hence we denote the eigenvalues of 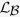 as 0 < *λ*_1_ < *λ*_2_ ≤ … ≤ *λ*_*n*_ and the corresponding normalised eigenvectors as ***v***_1_, ***v***_2,_ …, ***v***_*n*_. In essence, the spectra of 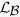 dictates the evolution of influenced consensus dynamics (3).

Controlling a consensus network via different leader agents impacts the network differently. The influence of an agent (or a group of agents) in a consensus network can be measured by the fluctuation of the network state ***x***(*t*) triggered by this agent(s). We shall characterise the state fluctuation by a differentiation operator.

**Figure 1:**
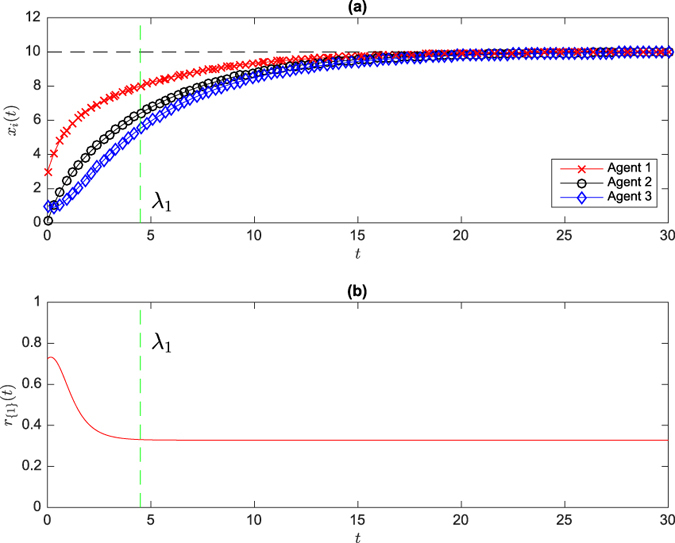
Coherence and tracking. (**a**) The trajectories of agents’ states of the influenced consensus network in Supplementary Figure 1 and (**b**) the trajectory of *r*_{1}_(*t*).

Taking the influenced consensus network in Supplementary Figure 1 as an example, we use quantity


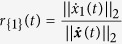


to characterise the influence of leader agent 1, where ||⋅||_2_ represents the Euclidean vector norm. The state evolution of each agent and *r*_{1}_(*t*) are shown in Fig. 1(a) and (b), respectively. The time window can be divided into two stages according to the convergence of *r*_{1}_(*t*), denoted by the coherence stage and the tracking stage, respectively. During the coherence stage, agents aggregate their states under the attractive force generated by diffusive couplings amongst agents. Since the consensus network is influenced by an external input *u* = 10, all agents subsequently track the external input via the leader agents. The two panels are both divided by the green dashed vertical lines at the time step *t* ≈ 4.5, on which *r*_{1}_(*t*) has converged to a steady value of 0.328. The coherence stage is governed by all the eigenvalues of 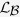 and the corresponding eigenvectors, but the tracking stage is dominated by *λ*_1_ and ***v***_1_. According to the Perron– Frobenius theorem, the entries of ***v***_1_ are all nonzero and can be selected to be positive^20^.

For a set of agents 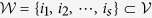, we define its *tempo centrality* (TC), denoted by 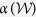, as 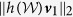, where ***v***_1_ is the normalised eigenvector corresponding to the smallest eigenvalue of 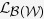, 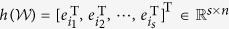, and 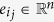 denotes the column vector with 1 for the *i*_*j*_-th entry and 0 otherwise, where *j* ∈ {1, 2, …, *s*}. It has been shown in ref. 21 that the long-term behaviour of


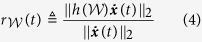


is determined by ***v***_1_, namely,


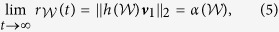


implying that 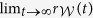 is independent of both the initial state ***x***(0) and the external input ***u***, as shown in Supplementary Figs 2 and 3. Note that TC takes values from entries of a positive normalised eigenvector, therefore 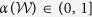. The TC characterises the tempo of agents with respect to that of the entire network in the tracking stage when they act as leaders.

### Computation of TC based on Temporal Data

The evolution of the network state is dictated by the network topology and the individual node dynamics. According to (5), the network snapshots that are generated by the individual dynamics can provide an alternative way for the computation of TC even when the network topology is unknown. We shall utilize this property for the design of an algorithm for computing TC from the temporal data of a consensus network.

Consider a consensus network with the set of leader agent 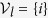 and the corresponding perturbed Laplacian matrix 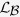. Denote by *h*_*i*_ and *h*_*j*_ as selection matrices for the leader agent {*i*} and a distinct agent {*j*}, respectively. The following result has been established linking the network state and the spectra of perturbed Laplacian matrix in ref. 21,


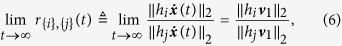


where ***v***_1_ is the normalized eigenvector corresponding to the smallest eigenvalue of 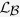.

We now show how TC can be computed from the network snapshots. Without loss of generality, designate agent 1 as the leader agent in the network, i.e., 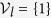. Discretizing (3) at the sampling points *t*_0_ ≤ *δ*_1_ < *δ*_2_ < … < *δ*_*m*_ yields an *m*-length snapshots of the consensus network, i.e., 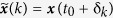 where *t*_0_ ≥ 0 is the initial time step and *k* = 0, 1, …, *m*. Denote 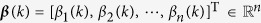 satisfying


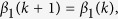



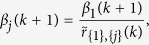


where


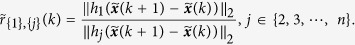


Then, it follows that


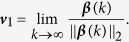


According to the definition, the first entry of 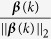 approaches the TC of agent 1 when *k* → ∞. A value threshold *ε* > 0 needs to be set such that the computation of 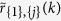 terminates when reaching its steady state. The above discussions conclude the following algorithm that computes the TC of leader agent 1.


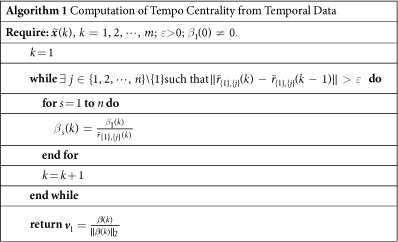


The complexity of Algorithm 1 depends on the convergence of *r*_{*i*},{*j*}_(*k*) for all 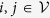, which is controlled by the *ε*. As a result, the computation complexity of Algorithm 1 is 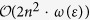 where *n* is the network size and *ω*(*ε*) denotes the total number of steps after which *r*_{*i*},{*j*}_(*k*) converges to its steady state with an accuracy of *ε*. Note that 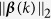 is a global information for a given influence structure and is usually not accessible to the agents in the network. However, according to (6), each agent can still infer the relative role of nodes via local measurements.

Here, we proceed to discuss the amount of data needed for the implementation of Algorithm 1. Theoretically, we have


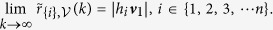


We thereby quantify the error of estimating ***v***_1_ with an *m*-length network snapshots 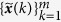 by


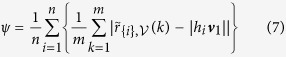


where


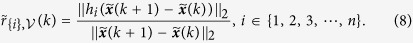


**Figure 2:**
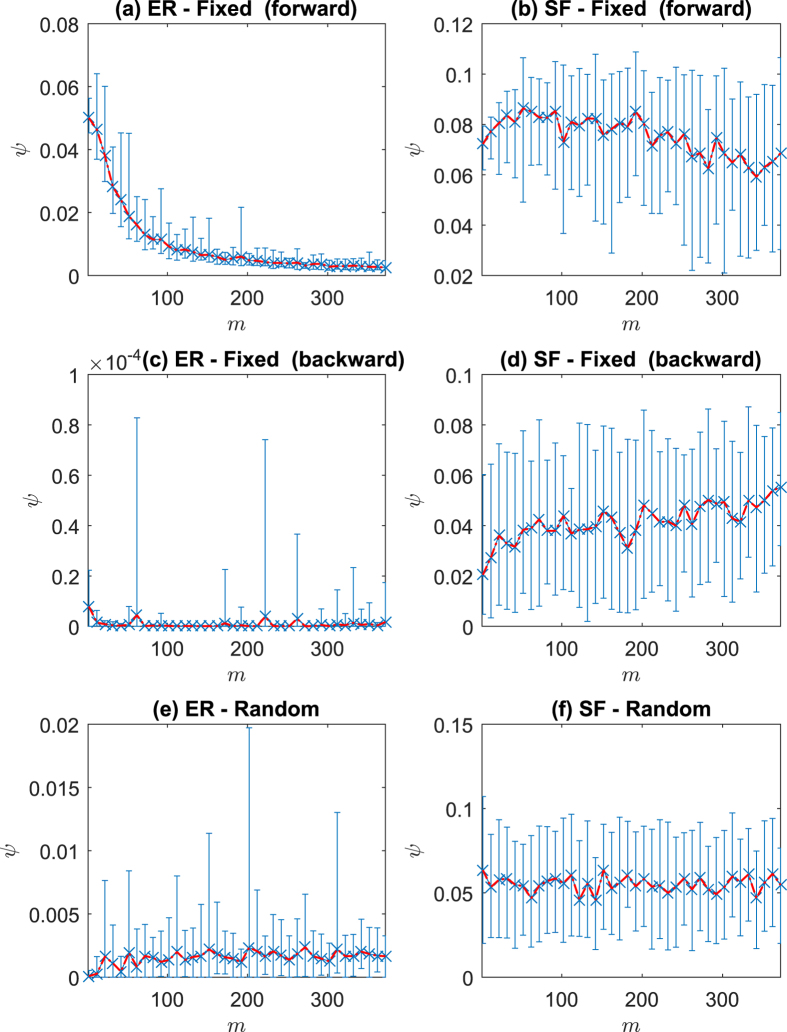
The error bar plot between the estimation error *ψ* and the length of snapshots *m* for forward sampling, backward sampling and random sampling in ER random networks (ER) and scale-free networks (SF) on 100 nodes. The error bar is plotted with 500 realizations for each network type and one leader is randomly selected for each such realizations. The edge occurrence probability for ER random networks is 0.05. In random sampling, at most *m* of snapshots are selected.

**Figure 3:**
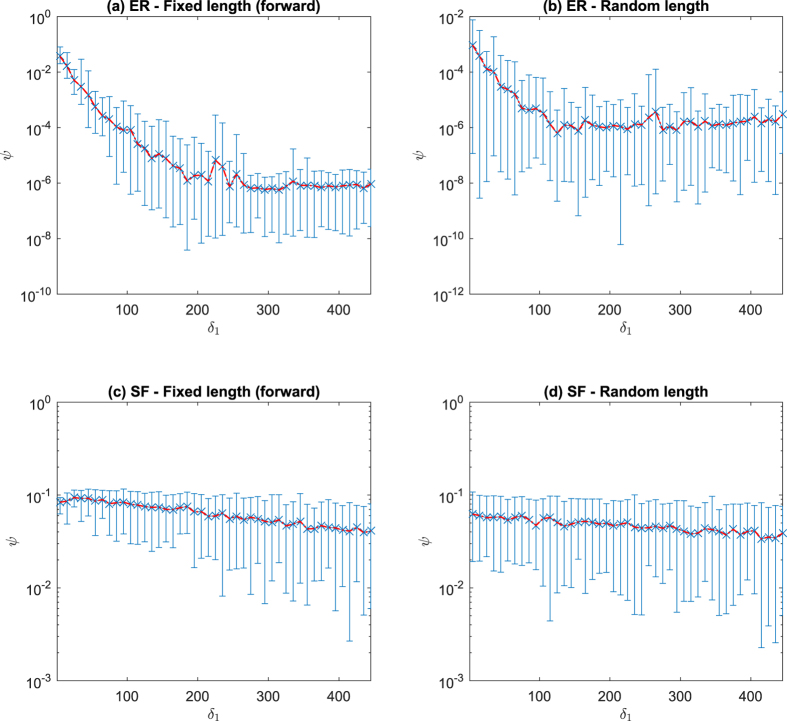
Error bar plot of the influence of initial point of sampling on *ψ* in 100-node ER random networks (ER) with forward sampling (**a**) and random sampling (**b**); and scale-free (SF) networks with forward sampling (**c**) and random sampling (**d**). A consecutive 20 snapshots from *δ*_1_ have been selected. 500 realizations are used for each type of network.

The smaller the value of *ψ* the more accurate the estimation of ***v***_1_ with network snapshots. Intuitively, more data will improve the estimation accuracy. Figure 2(a) and (b) show the estimation error *ψ* is decreasing when more snapshots are used in an ER random network and a scale-free network. In fact, we can start sampling the network state *x*(*t*), over a time interval of *t* ∈ [*t*_1_, *t*_2_], from *t*_1_ in a forward sequence (forward sampling), or from *t*_2_ in a reversed sequence (backward sampling). The backward sampling is more preferable since 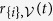 is closer to its steady state. In Fig. 2(c) and (d), we apply the backward sampling and the estimation error *ψ* is shown to be dramatically decreased compared with the forward sampling. In the backward sampling, it turns out that the length of snapshots *m* has little influence on the estimation error *ψ*. The two methods mentioned above sample *x*(*t*) with respect to time *t* uniformly, which is somewhat difficult to achieve due to the disturbance in measurement. We subsequently examine the performance of random sampling of *x*(*t*), namely, selecting at most *m* snapshots randomly. Figure 2(e) and (f) show that the performance of random sampling and the length of snapshots *m* also has little influence on the improvement of estimation accuracy in this scenario (for instance, in ER random networks, the estimation error obtained from *m* = 5 snapshots in random sampling is better than that from *m* = 300 in a forward sampling, as shown in Fig. 2(a) and (e)). We now show the influence of initial point of sampling *δ*_1_ on *ψ* in Fig. 3. It turns out that the later the sampling process starts the more accurate the effect of estimation. Supplementary Figures 16, 17 and 18 show this trend in more networks. Therefore, the initial point of sampling is a critical factor. As a result, we can conclude that (a) the careful selection of sampling points is critical in the efficiency of Algorithm 1, (b) the length of snapshots can be as short as *m* = 2 (in order to make the computation of (8) feasible) and (c) the sampling points can be heterogeneously distributed over the time interval.

### Correlation with Network Performance

In this section, we proceed to explore the correlation between TC and two network performance metrics, namely, the convergence rate of consensus and the network robustness, both of which are closely related to the spectra of 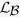 7,21. Since TC is derived from the normalised eigenvector of the perturbed Laplacian matrix, there are analytic connections between TC and network performances that are determined by the spectra of 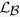. However, one can compute TC based on network snapshots and thus the related network performances can be estimated with a data-driven approach.

### Convergence Rate of Consensus

The smallest eigenvalue of 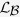 (denoted by *λ*_1_ in our discussion) provides us with the convergence rate of the consensus network influenced by the external input, on the other hand, *λ*_1_ reflects how fast the diffusion of the external input (friendly or malicious, deterministic or stochastic) proceeds on the network^7^,^22^. Therefore, *λ*_1_ can be regarded as a measure of spreading power of nodes in a consensus network from the perspective of the propagation efficiency of external inputs (refer to section 1 in Supplementary Information for details). Here, we provide some analytical facts between TC and *λ*_1_. For an arbitrary agent 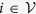 with the corresponding selection matrix *h* and influence matrix 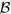, starting from


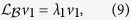


and multiplying (9) by **1**^*T*^ from left on both sides yields


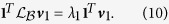


Since the entries of ***v***_1_ can be chosen to have the same signs,


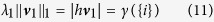


where 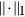 represents the 1-norm of vectors.

**Figure 4:**
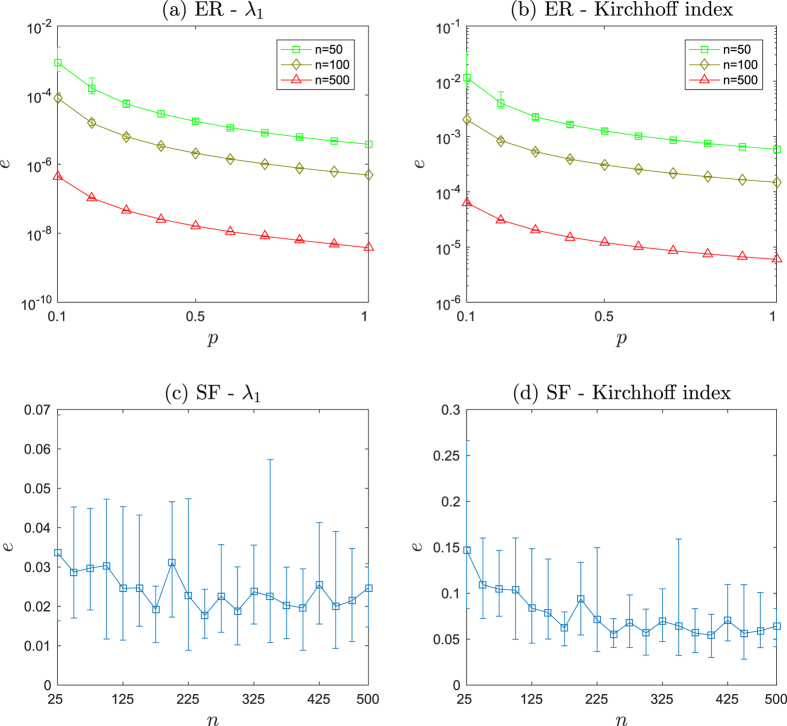
Error bar plot of approximation error in ER random networks and scale-free networks. The error of approximating *λ*_1_ by 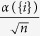 in ER random networks (**a**) and scale-free networks (**c**); the error of approximating Kirchhoff index by 
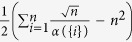
 in ER random networks (**b**) and scale-free networks (**d**). The possibility for edge occurrence *p* ranges from 0.1 to 1 at the regular interval 0.1 (the plot for *p* = 0.05 to *p* = 0.1 is shown in Supplementary Figure 19). For each realization of the network, we compute the TC for all nodes 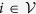 in the network and the corresponding 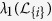. Since the Kirchhoff index corresponds to the entire network, we only need to compute the Kirchhoff index for each realization of the network and its approximation by TC. 500 realizations are used for each type of network.

**Figure 5:**
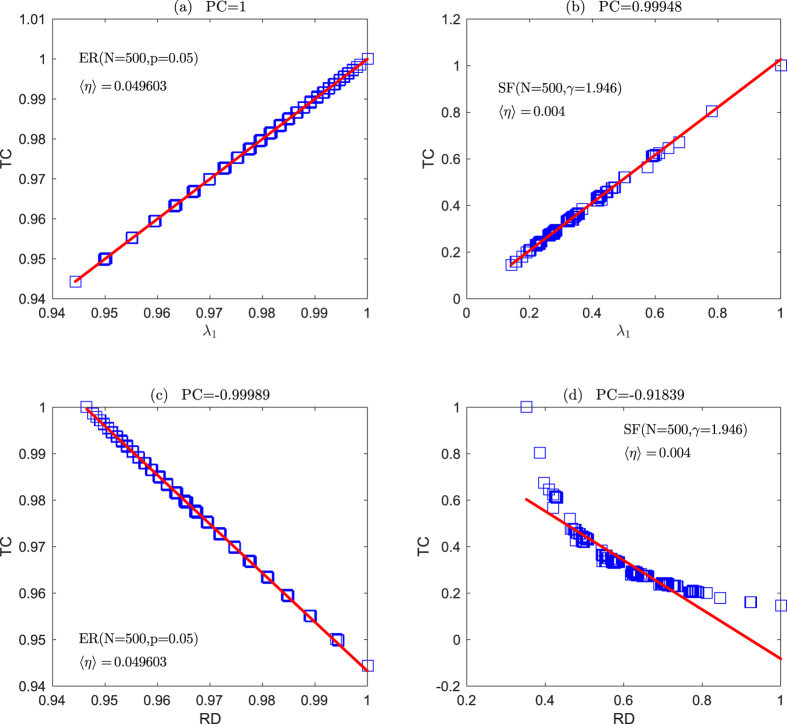
The TC as a function of *λ*_1_ in an Erdős-Rényi (ER) random network (**a**) and a scale-free network (SF) (**b**). The Pearson coefficient between TC and *λ*_1_ are 1 (ER) and 0.99948 (SF), respectively. The TC as a function of resistance distance (RD) in an Erdős-Rényi (ER) random network (**c**) and a scale-free network (SF) (**d**). The Pearson coefficient between TC and RD are −0.99989 (ER) and −0.91839 (SF), respectively.

Note that ***v***_1_ is normalised in terms of the 2-norm of vectors, i.e., 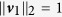. According to the equivalency of vector norms, we have


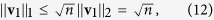


which implies the propagation efficiency *λ*_1_ is lower bounded by 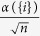. Figure 4(a,c) and Supplementary Table 3 show that *λ*_1_ can be well-approximated by 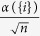 in ER random networks, scale-free networks and many empirical networks. The error bar of approximation errors in Fig. 4 reflects that the approximation effect is improved in large and dense networks. However, as shown in Supplementary Figures 19(a) and (c), the maximum relative errors corresponding to large sparse networks still imply an accurate estimation of *λ*_1_ with TC. We proceed to use Pearson correlation analysis to show that there is a strong linear correlation between TC and *λ*_1_. The Pearson coefficient between TC and *λ*_1_ in an 500-node Erdős-Rényi (ER) random network and a 500-node scale-free network (SF) is shown in Fig. 5(a,b). The Pearson coefficients (PC) are 1 and 0.99943 for ER random network and scale-free network, respectively, implying the total positive correlation between TC and *λ*_1_.

### Kirchhoff Index and Network Robustness

Viewing each edge in the network as a one-Ohm resistor, the effective resistance from the leader agents to all agents in the network is shown to be an index of network robustness from the perspective of both the 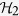 system norm (ability of disturbance rejection in the presence of additive noise in consensus networks)^23^,^24^ and the vulnerability of the network connectivity to node or edge failures^23^,^24^,^25^. The perturbed Laplacian matrix turns out to be an important construct that is closely related to the effective resistance. Specifically, the 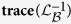 measures the functional robustness of the influenced consensus network in terms of the variance of deviation from consensus and the disturbance rejection properties of consensus networks, both of which can be characterised by the effective resistance of the network^22^,^26^,^27^,^28^,^29^. The structural robustness of complex networks has also been widely investigated in the context of percolation theory^30^,^31^.

The resistance distance from agents *i* to *j*, denoted by *d*_*ij*_, is the effective resistance between *i* and *j*, which can be quantified by the diagonal entries of 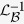
24,32. Suppose that the network is influenced by a single external input via agent 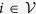 and denote the resultant perturbed Laplacian matrix as 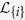, which is invertible if 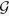 is connected^32^. The resistance distance from input *u* to agent 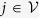 is 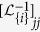
22,32, and thus the resistance distance from the agent *i* to agent *j* is such that 
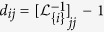
. Hence, the resistance distance from agent *i* to all agents in 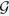, denoted by *q*_*i*_, is the summation of *d*_*ij*_ over all 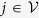, i.e.,


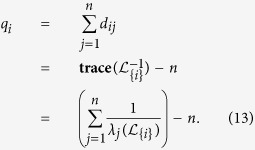


In the following discussion, we shall refer to *q*_*i*_ as the resistance distance (RD) index of 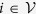. The Kirchhoff index of a network 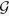 (introduced by Klein and Randic′ in ref. 24) is half of the total effective resistance between all pair of nodes in 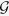 and can therefore be computed by


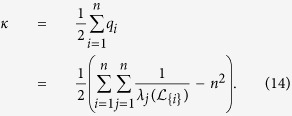


Kirchhoff index is a measure of connectivity and size of a network in terms of resistance distance^23^. A network with smaller value of Kirchhoff index is considered to be more robust to node or edge failures. Kirchhoff index is also related to the average power dissipation of the circuit with a random current excitation and the average commute time of a Markov chain on a graph. Algorithms have also been proposed for minimizing the Kirchhoff index a network^33^.

The smallest eigenvalue of the perturbed Laplacian matrix can be considered as the perturbation of the smallest eigenvalue of the Laplacian matrix, which is always zero. The influence of this perturbation decreases when the network size grows, and consequently the term 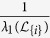 would dominate *q*_*i*_ in (13). Based on the estimation of 
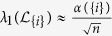
, we can further approximate *q*_*i*_ by


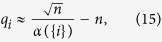


and the Kirchhoff index by


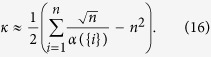


**Figure 6:**
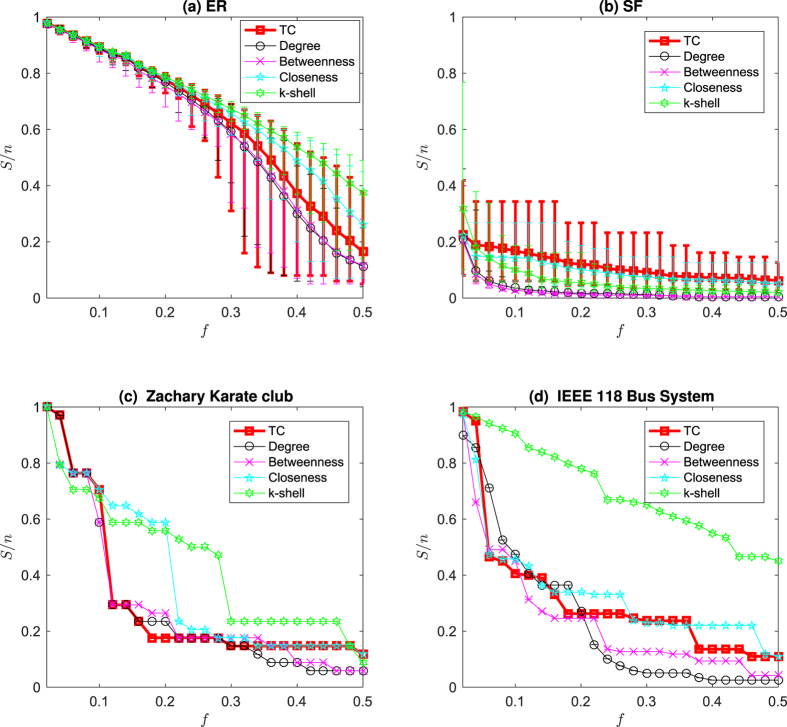
The effect of centrality attack 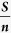 as a function of the fraction of removed nodes *f* for centrality metrics including TC, degree, betweenness, closeness and k-shell. (**a**) The ER random (ER) network with *n* = 500 and *p* = 0.05, (**b**) the scale-free (SF) network with *n* = 100 and 〈*η*〉 = 0.02, (**c**) the Karate club network with *n* = 34, and (**d**) the IEEE 118 bus system network with *n* = 118. The error bar plots for ER and SF are over 500 realizations for each network type.

Figure 4(b) and (d) and Supplementary Table 3 show the effect of this approximation in ER random networks, scale-free networks, and empirical networks. The maximum relative errors shown in Supplementary Figures 19(c) and 19(d) corresponding to large sparse networks still imply a good estimation of Kirchhoff index with TC. The Pearson coefficient between TC and resistance distance in 500-node Erdős-Rényi (ER) random network and 500-node scale-free network is shown in Fig. 5(c) and (d). The Pearson coefficients (PC) are −0.99988 and −0.91485 for the ER random network and the scale-free network (both implying the strong negative correlations). We have shown the results of Pearson correlation analysis and the corresponding regression coefficient in Supplementary Tables 1 and 2 for all 7 metrics mentioned in this paper under ER random networks, scale-free networks, and empirical networks. It has been shown that there exist a total positive correlation between TC and *λ*_1_, and a strong negative correlation between TC and resistance distance for different classes of networks, enabling us to estimate these network performance metrics from network snapshots. The robustness of nodes in terms of centrality attack is shown in Fig. 6 and Supplementary Figures 21 and 22.

### Zachary’s Karate Club

**Figure 7:**
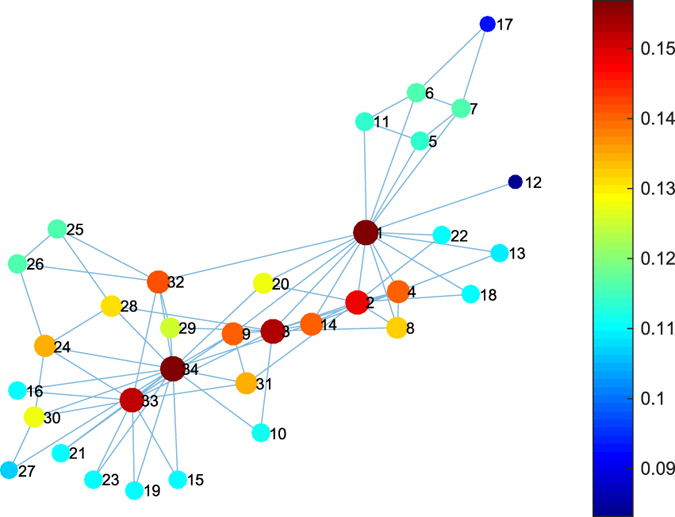
The TC distribution on Zachary’s Karate club network. The size of dots are proportional to their TC value. The top two largest TC values are achieved for agent 34 and agent 1 (0.1569 and 0.1561, respectively).

As a case study, we have applied TC to quantify the role of members in the Zachary’s Karate club^34^. In this case, TC highlights the president (node 1) and lesson instructor (node 34) in the club by assigning the top two largest TC values to them. The rank of each node in the network in terms of the value of TC, degree, eigenvector, betweenness, closeness and k-shell is shown in Table 1. We compute the Spearman’s rank correlation coefficient (the Pearson correlation between the rank values of two variables) between TC and degree, eigenvector, betweenness, closeness and k-shell, respectively. TC is shown closer to the degree (with Spearman’s rank correlation coefficient as 0.9422) in this example, followed by closeness (0.9251), betweenness (0.9013), eigenvector (0.8597) and k-shell (0.8481). We subsequently compared the TC with above five centrality metrics in the Karate club network by normalising the values of these centrality metrics as illustrated in Supplementary Figure 4. The distribution of the TC values in the heat map is shown in Fig. 7. According to the pattern of the normalised centrality values, TC identifies the distinct role of nodes compared with the other five centrality metrics in this example. The scatter plots and Pearson coefficient between TC and seven other metrics (above five centralities plus *λ*_1_ and RD) in Karate club network are shown in Supplementary Figures 5 and 6 respectively. More scatter plots are shown in Supplementary Figures 7–15 for 9 empirical networks.

## Discussion

Instead of quantifying the importance of a single agent in the network, the importance of a group of agents can also be characterised by TC, which is meaningful when the group is considered as in union or multiple leader agents are needed to exert influence on a consensus network. For a given group of leader agents, the larger the value of TC the faster the external influence propagates throughout the network and the less resistance the external influence has to overcome. In the meanwhile, the larger TC implies that the leader agent related to the influence matrix evolves towards the external input with a faster tempo than the rest of the network. Note that the influence of a single agent decreases when the network size grows; as a result, more leader agents are needed to exert a stronger influence.

Topology identification (through such methods as compressive sensing, linear system identification, power spectral analysis, convex programming etc.) is generally based on the minimization of the distance between the estimated and the real network structures. Therefore, the entry-wise errors are acceptable for the optimization procedure. However, the role of an individual node can be different with respect to an entry-wise error, even when the two estimated network structures are equivalent from an optimization perspective. Our data-driven approach is robust to this ambiguity.

The second smallest eigenvalue of the graph Laplacian is known as the algebraic connectivity of a network, characterising the convergence rate of the consensus process^10^,^35^; and the corresponding eigenvector, called the Fiedler vector, has been widely applied in the network partitioning^35^. We can regard all agents in a network 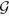 along with the set of external input 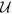 as an expanded directed graph, in which the directed edges are present only from the external input to the leader agents. The vector 
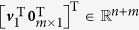
 can be considered as the Fiedler vector for this expanded digraph, where **0**_*m*×1_ denotes the *m* × 1 vector with all zero entries. The impact of the external input on the network 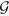 can be characterised by the spectra of 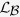. Using the spectral decomposition of the perturbed Laplacian matrix, i.e., 
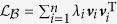
, we observe that the eigenvector ***v***_1_ shapes the behaviour of the network when the dynamics in directions of ***v***_2_, ***v***_3_ …, ***v***_*n*_ vanish. As one can see from Supplementary Figure 20, that the distribution of entries in ***v***_1_ is shaped by the placement of the leader agent, that is, the farther a node is from the leader agent, the larger the absolute value of its corresponding entry in ***v***_1_.

A summary of the utility of TC and our proposed approach is as follows. The TC is shown to be closely correlated to two performance metrics for consensus networks, namely, the convergence rate of the influenced consensus (characterising the propagation efficiency of the external influence over a consensus network) and the network functional robustness in terms of disturbance rejection; the TC can be computed from the network snapshots even when the network topology is unknown; the data-driven approach in this paper enables each agent to determine its relative role in the network by using the state of its neighbors; this relative role obtained by each agent via local information is a global information; since the linearization of the Kuramoto dynamics around the origin is consensus dynamics the results discussed in this paper have a natural extension to the Kuramoto model, widely investigated for oscillator networks^36^.

## Methods

### Normalization of Centrality

The centrality vector ***c*** = [*c*_1_, *c*_2_, …, *c*_*n*_]^Τ^ of a network 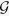 is composed of the centrality value *c*_*i*_ of all nodes 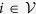 in 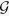. Since the value of centrality could differ in scale, we normalize *c*_*i*_ to the interval [0, 1] by the maximum value of a given centrality as follows


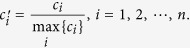


### Pearson Correlation Coefficient

The Pearson correlation coefficient for two vectors 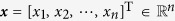 and 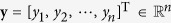 is defined as


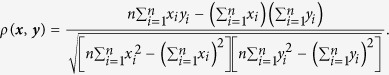


### Relative Error

The approximation error is measured by the relative error between vectors. Let 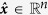 be an approximation of vector 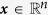. The relative error used in this paper is,


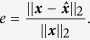


### Edge Density

The edge density of a network 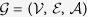 is defined as the ratio between the number of edges and the maximum number of possible edges in a network, i.e.,


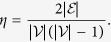


### Error Bar

The error bar plots in this paper represent the fluctuation of data around their respective mean. The marker in the centre of an error bar represents the mean of the data; the upper and lower intervals represent the deviation of the mean from the maximum and minimum value of the data.

### Centrality Attack

In the centrality attack, the nodes in a network are sorted in descending order according to their centrality value, then the first *f* ∈ (0, 1] fraction of nodes are removed from the network. The normalized size of largest connected component 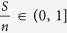 is used to quantify the effect of the attack.

### Networks

An *n*-node Erdős-Rényi (ER) random network with the probability of edge realization *p* ∈ [0, 1] is denoted by 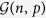 and is constructed by randomGraph.m in Octave routines for network analysis^37^. The scale-free network with degree distribution *p*(*k*) ~ *k*^−*γ*^ is generated by SFNG.m in B-A Scale-Free Network Generation and Visualization^38^. The empirical networks are from KONECT^39^. The centrality value of the network degree, eigenvector, betweenness and closeness are computed using Octave routines for network analysis^37^. The k-shell is computed by corenums.m in Graph Algorithms In Matlab Code (gaimc)^40^.

## Additional Information

**How to cite this article:** Shao, H. *et al*. Inferring Centrality from Network Snapshots. *Sci. Rep.* **7**, 40642; doi: 10.1038/srep40642 (2017).

**Publisher's note:** Springer Nature remains neutral with regard to jurisdictional claims in published maps and institutional affiliations.

## Supplementary Material

Supplementary Material

## Figures and Tables

**Table 1:** Rank of each node in Zachary’s Karate club network in terms of TC, degree (D), eigenvector (E), betweenness (B), closeness (C) and k-shell (K).

Node	TC	D	E	B	C	K	Node	TC	D	E	B	C	K
**1**	2	2	2	1	1	1	**18**	30	28	24	29	22	28
**2**	5	5	5	7	9	2	**19**	24	29	18	30	29	29
**3**	3	4	3	4	2	3	**20**	15	19	13	9	8	15
**4**	9	6	8	15	10	4	**21**	28	30	19	31	30	30
**5**	21	17	28	21	20	11	**22**	29	31	23	32	23	31
**6**	17	11	26	10	17	12	**23**	25	32	20	33	31	32
**7**	18	12	27	11	18	13	**24**	10	10	12	13	16	16
**8**	12	13	11	23	14	5	**25**	20	20	32	18	24	17
**9**	7	8	6	6	5	6	**26**	19	21	31	16	25	18
**10**	23	23	17	20	15	23	**27**	32	33	30	34	33	33
**11**	22	18	29	22	21	14	**28**	13	14	15	12	11	19
**12**	34	34	33	24	32	34	**29**	16	22	16	19	13	20
**13**	31	24	25	25	26	24	**30**	14	15	14	17	19	21
**14**	8	9	7	8	6	7	**31**	11	16	10	14	12	8
**15**	27	25	21	26	27	25	**32**	6	7	9	5	4	22
**16**	26	26	22	27	28	26	**33**	4	3	4	3	7	9
**17**	33	27	34	28	34	27	**34**	1	1	1	2	3	10

The rank is arranged in descending order according to the respective centrality value.
