# Supplementary material for: Inferring Centrality from Network Snapshots
Source: Sci Rep. 2017 Jan 18;7:40642. doi: 10.1038/srep40642 (PMC5241648; doi:10.1038/srep40642)
Supplement: Supplementary Material [file srep40642-s1.pdf]

Supplementary Material of "Inferring Centrality from Network Snapshots"

Haibin Shao, Mehran Mesbahi, Dewei Li, and Yugeng Xi

1 Convergence rate of influenced consensus

Examining the solution to

$$\dot{\boldsymbol{x}}(t) = -\mathcal{L}_{\mathcal{B}}\boldsymbol{x}(t) + \mathcal{B}\boldsymbol{u}$$

yields

$$\begin{aligned} \boldsymbol{x}(t) &= e^{-\mathcal{L}_{\mathcal{B}}t}\boldsymbol{x}(0) + \int_0^t e^{-\mathcal{L}_{\mathcal{B}}(t-\tau)}\mathcal{B}\boldsymbol{u}d\tau \\ &= Ve^{\Lambda t}V^T\boldsymbol{x}(0) + V\left(\int_0^t e^{\Lambda(t-\tau)}d\tau\right)V^T\mathcal{B}\boldsymbol{u} \\ &= T_1 + T_2 \end{aligned}$$

where $V \in \mathbb{R}^{n\times n}$ is composed of the normalized eigenvectors of $\mathcal{L}_{\mathcal{B}}$,

$$T_1 = V\mathbf{diag}\left\{e^{-\lambda_1(\mathcal{L}_{\mathcal{B}})t},\, e^{-\lambda_2(\mathcal{L}_{\mathcal{B}})t}, \cdots,\, e^{-\lambda_n(\mathcal{L}_{\mathcal{B}})t}\right\}V^T\boldsymbol{x}(0)$$

and

$$T_2 = V\mathbf{diag}\left\{\frac{1}{\lambda_1}(e^{-\lambda_1(\mathcal{L}_{\mathcal{B}})t}-1),\, \frac{1}{\lambda_1}(e^{-\lambda_1(\mathcal{L}_{\mathcal{B}})t}-1), \cdots,\, \frac{1}{\lambda_n}(e^{-\lambda_n(\mathcal{L}_{\mathcal{B}})t}-1)\right\}V^T\mathcal{B}\boldsymbol{u}.$$

The propagation efficiency in our work means the rate at which the external influence propagates through a consensus network. In this sense, $\lambda_1(\mathcal{L}_{\mathcal{B}})$ serves as a lower bound of convergence rate of consensus to the external influence (in the case of homogeneous input) for all the agents in an influenced consensus network.

2 Supplementary Figures

2.1 An influenced consensus network

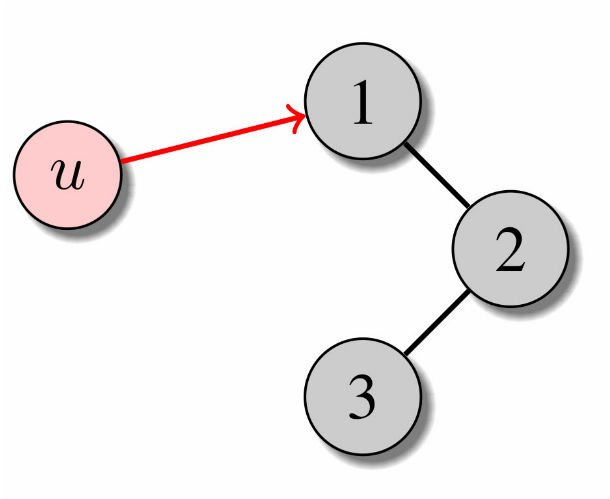

Figure 1: The structure of an influenced consensus network.

2.2 The influence of $\boldsymbol{x}(0)$ and $\boldsymbol{u}$ on $\lim_{t\to\infty} r_{\mathcal{W}}(t)$

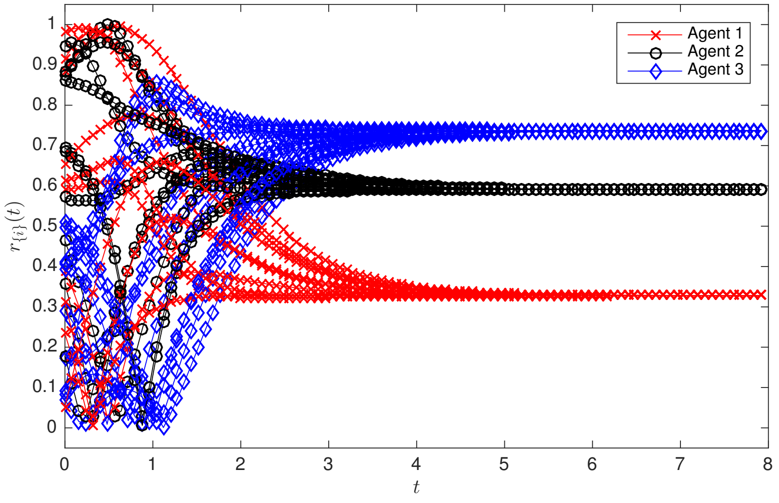

Figure 2: The trajectories of $r_{\{i\}}(t)$ for each agent $i \in \{1, 2, 3\}$ in the influenced consensus network shown in Figure 1. The network has been initiated from 10 different initial conditions.

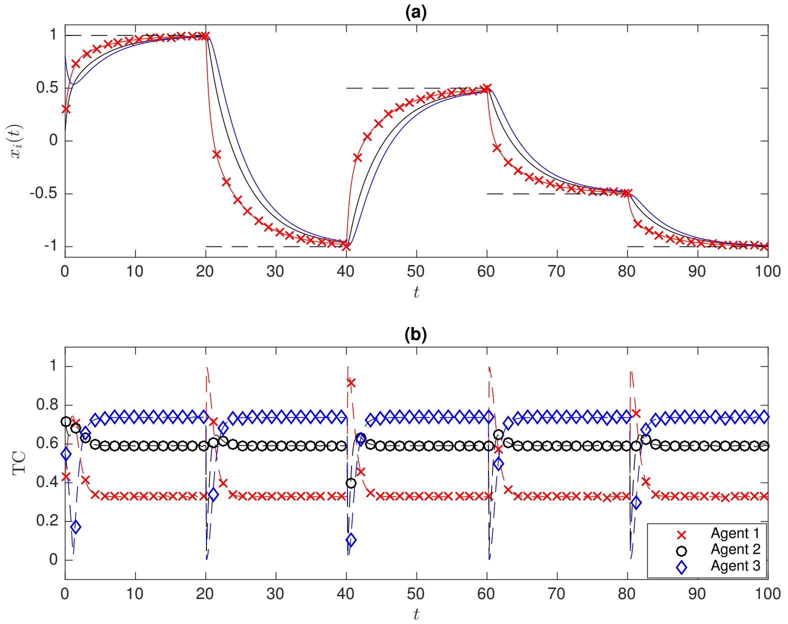

Figure 3: Equilibrium shifting in the influenced consensus network shown in Figure 1. (a) the trajectory of agents with $u = 1$ for $t \in [0, 20]$, $u = -1$ for $t \in [20, 40]$, $u = 0.5$ for $t \in [40, 60]$, $u = -0.5$ for $t \in [60, 80]$ and $u = -1$ for $t \in [80, 100]$. (b) the corresponding trajectories of $r_{\{i\}}(t)$ for each agent $i \in \{1, 2, 3\}$.

2.3 The normalized centrality value in Zachary's Karate club network

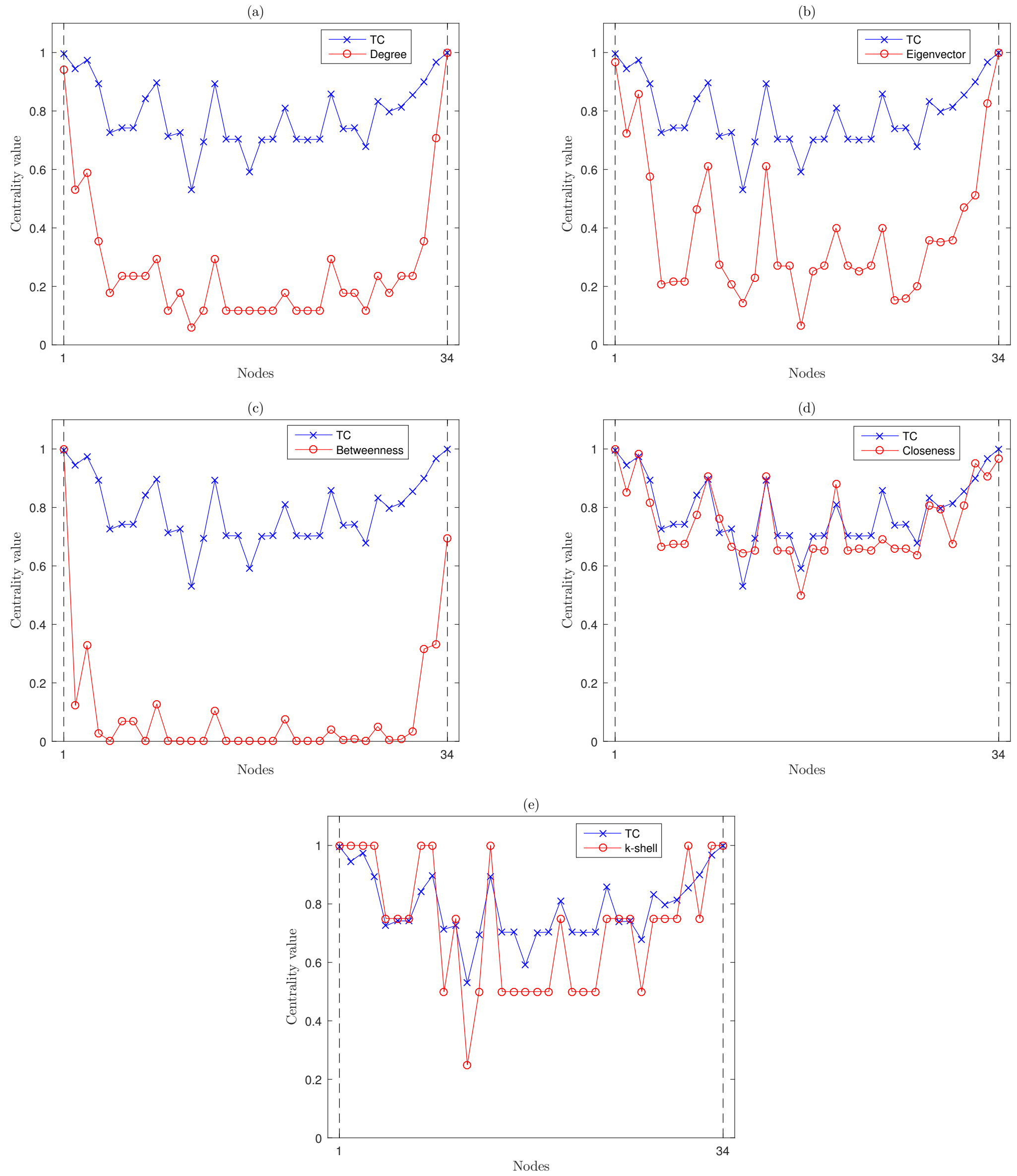

Figure 4: The normalized centrality value in Zachary's Karate club network. (a) TC versus degree. (b) TC versus eigenvector. (c) TC versus betweenness. (d) TC versus closeness. (e) TC versus k-shell. Note that the closeness and k-shell are closer to TC compared to the other three centrality metrics.

2.4 Karate club network

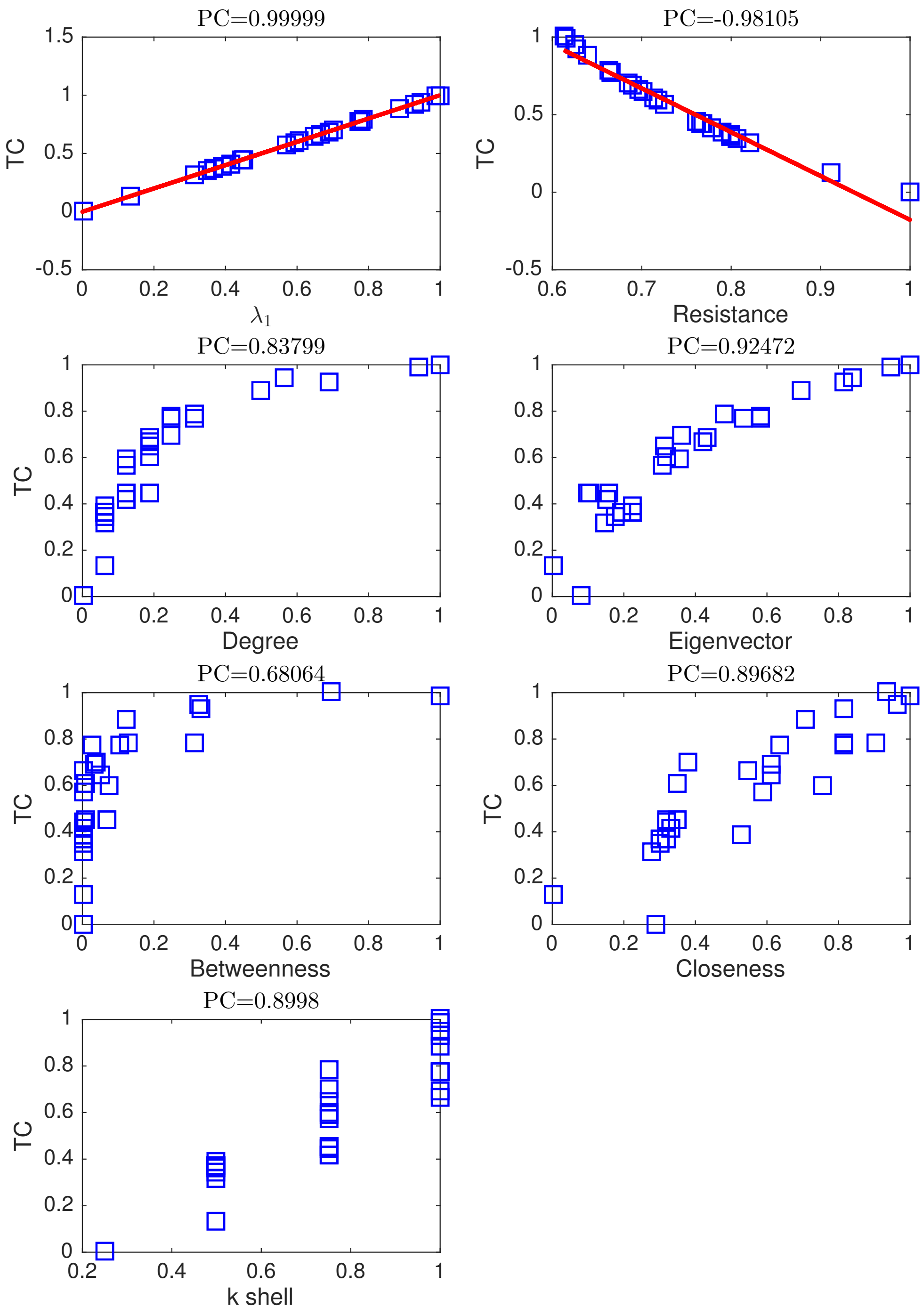

Figure 5: TC as a function of λ_1, resistance distance (RD), degree, eigenvector centrality, betweenness, closeness and k-shell with the corresponding Pearson coefficient (PC) in the Karate Club network.

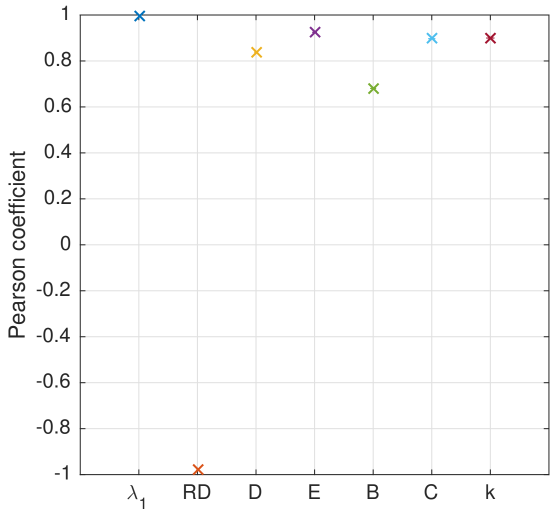

Figure 6: Pearson coefficient between TC and λ_1, resistance distance (RD), degree (D), eigenvector (E), betweenness (B), closeness (C) and k-shell (k) in the Karate Club network.

2.5 Scatter plots for empirical networks

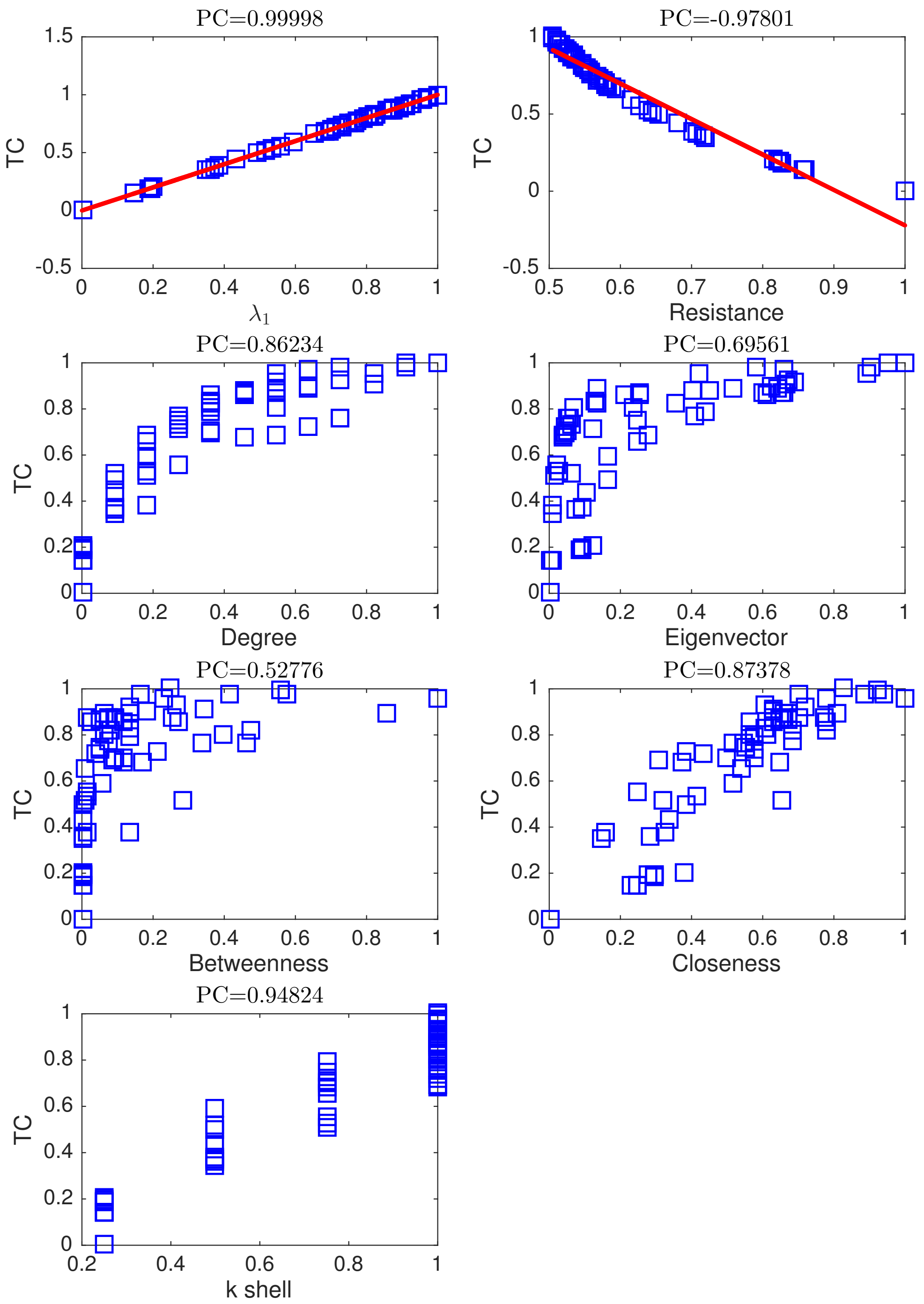

Figure 7: TC as a function of λ_1, resistance distance (RD), degree, eigenvector centrality, betweenness, closeness and k-shell with corresponding Pearson coefficient (PC) in Dolphins network.

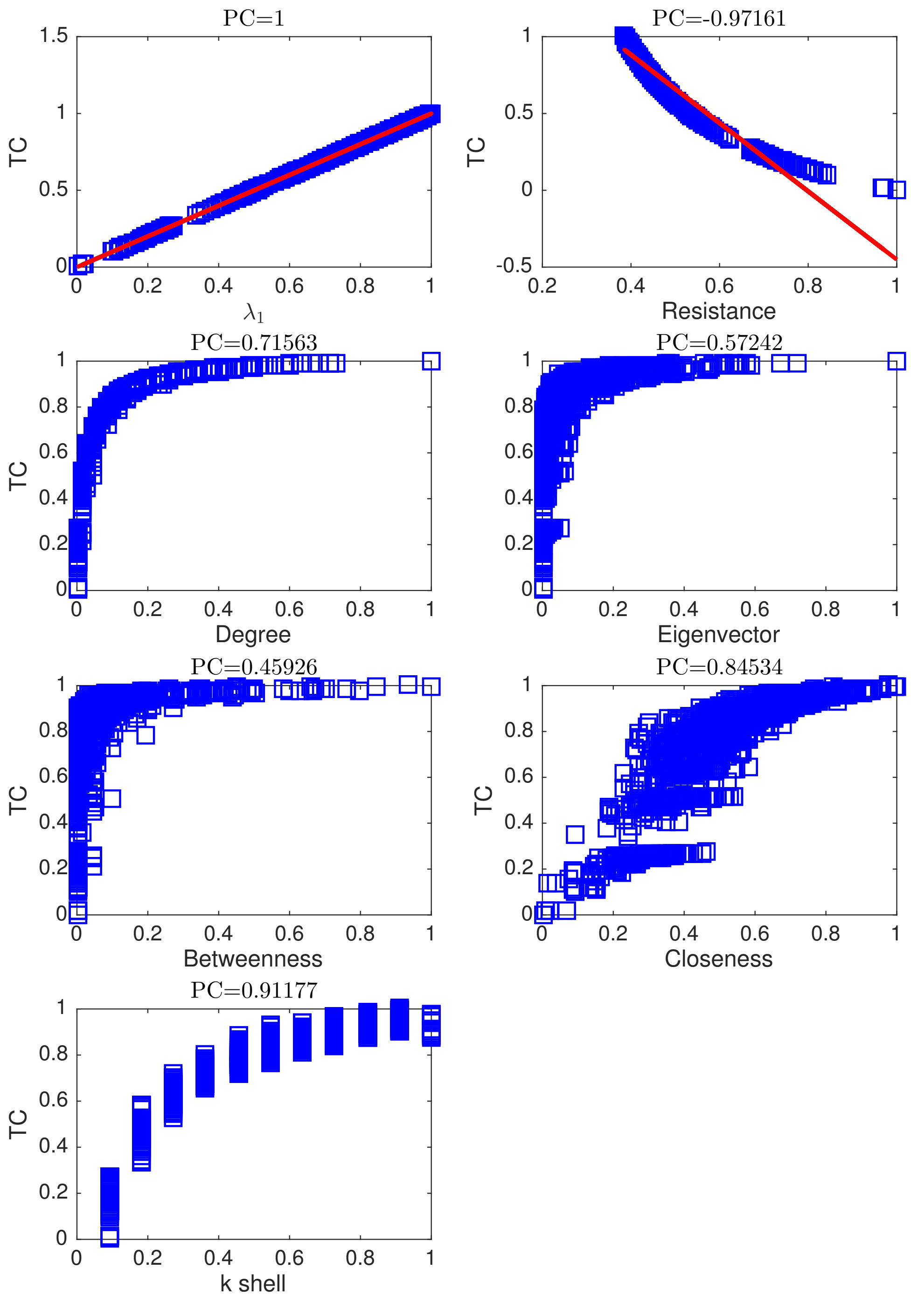

Figure 8: TC as a function of λ_1, resistance distance (RD), degree, eigenvector centrality, betweenness, closeness and k-shell with corresponding Pearson coefficient (PC) in U. Rovira i Virgili network.

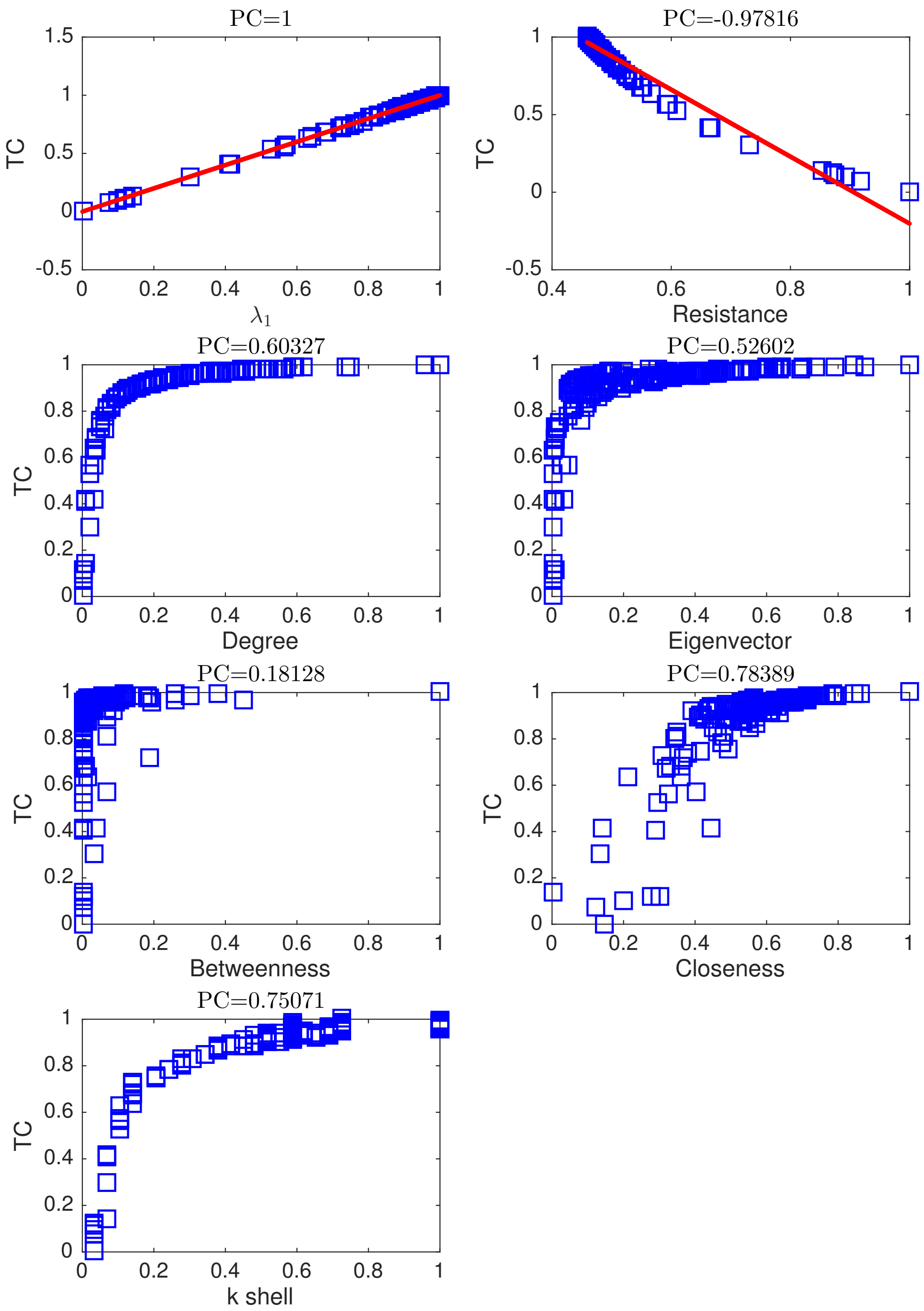

Figure 9: TC as a function of λ_1, resistance distance (RD), degree, eigenvector centrality, betweenness, closeness and k-shell with corresponding Pearson coefficient (PC) in Jazz musicians network.

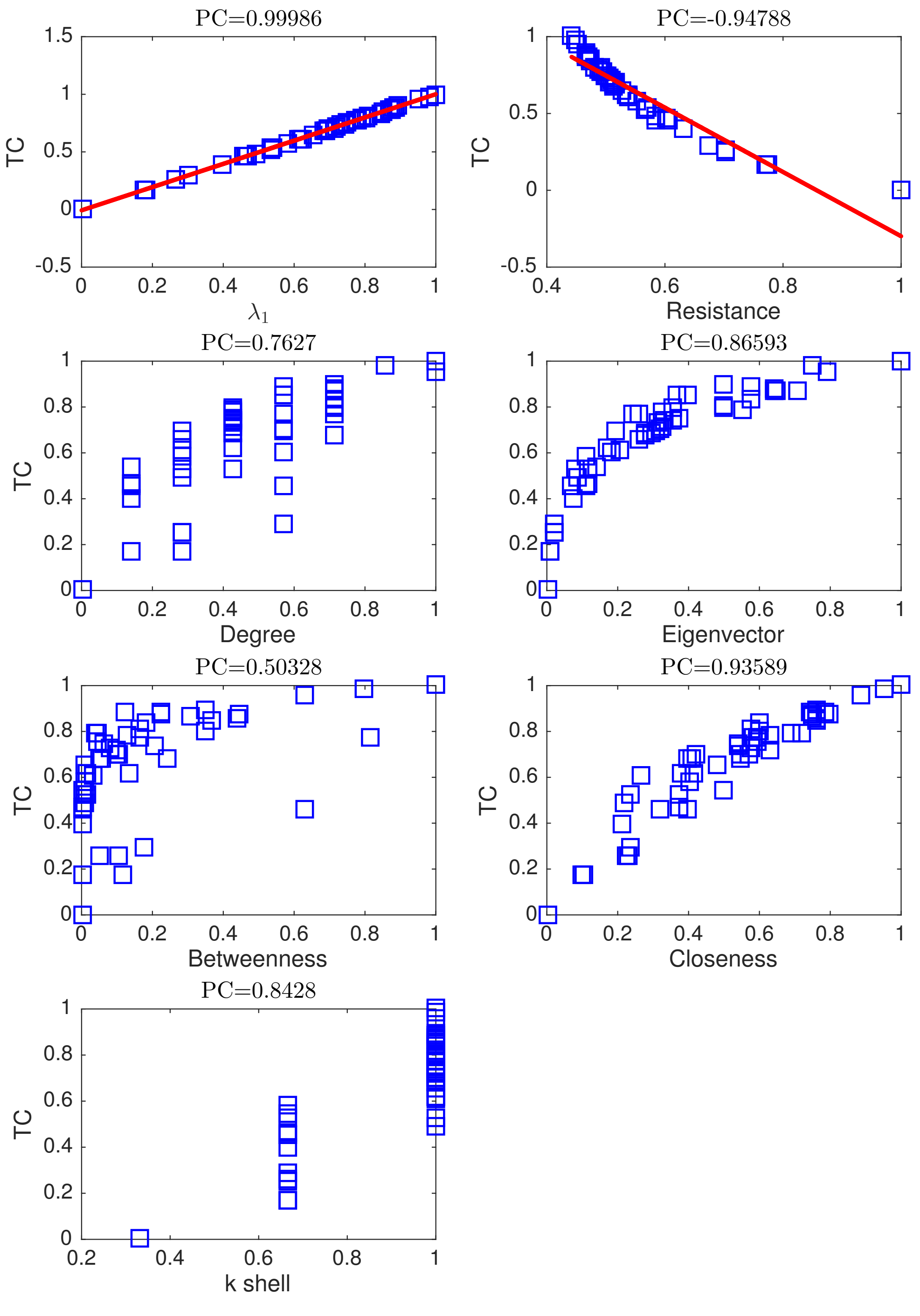

Figure 10: TC as a function of λ_1, resistance distance (RD), degree, eigenvector centrality, betweenness, closeness and k-shell with corresponding Pearson coefficient (PC) in Contiguous USA network.

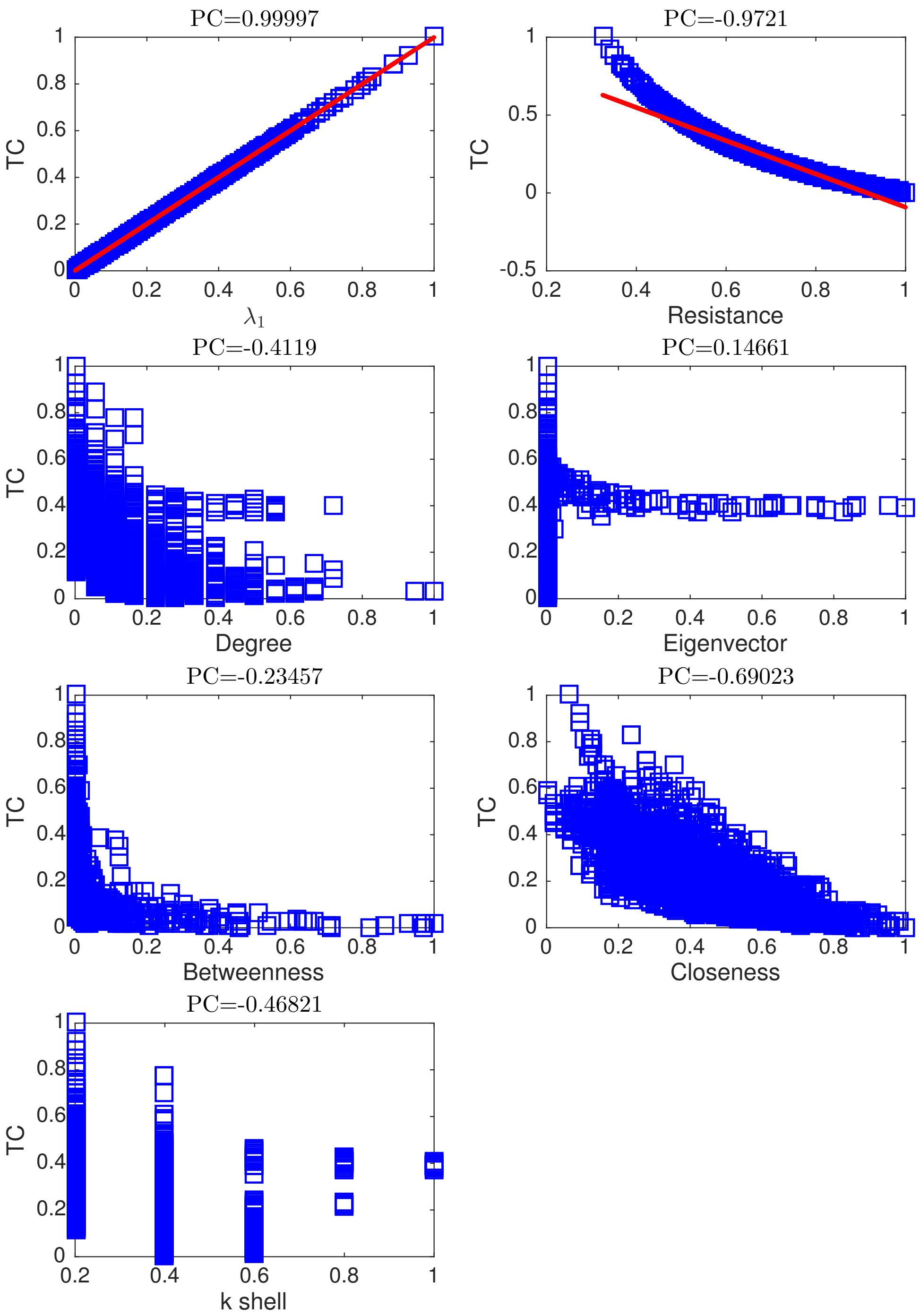

Figure 11: TC as a function of λ_1, resistance distance (RD), degree, eigenvector centrality, betweenness, closeness and k-shell with corresponding Pearson coefficient (PC) in US power grid network.

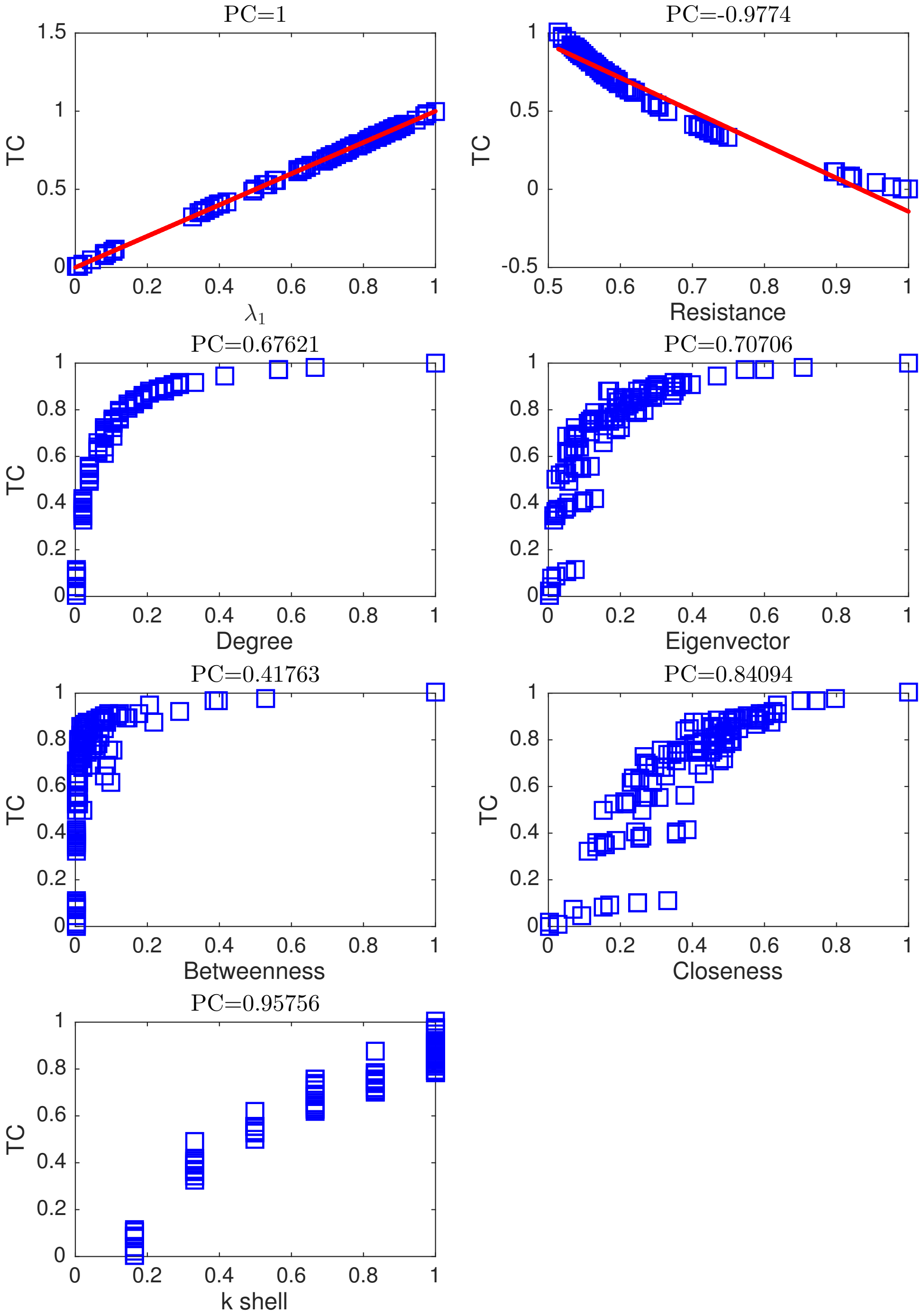

Figure 12: TC as a function of λ_1, resistance distance (RD), degree, eigenvector centrality, betweenness, closeness and k-shell with corresponding Pearson coefficient (PC) in David Copperfield network.

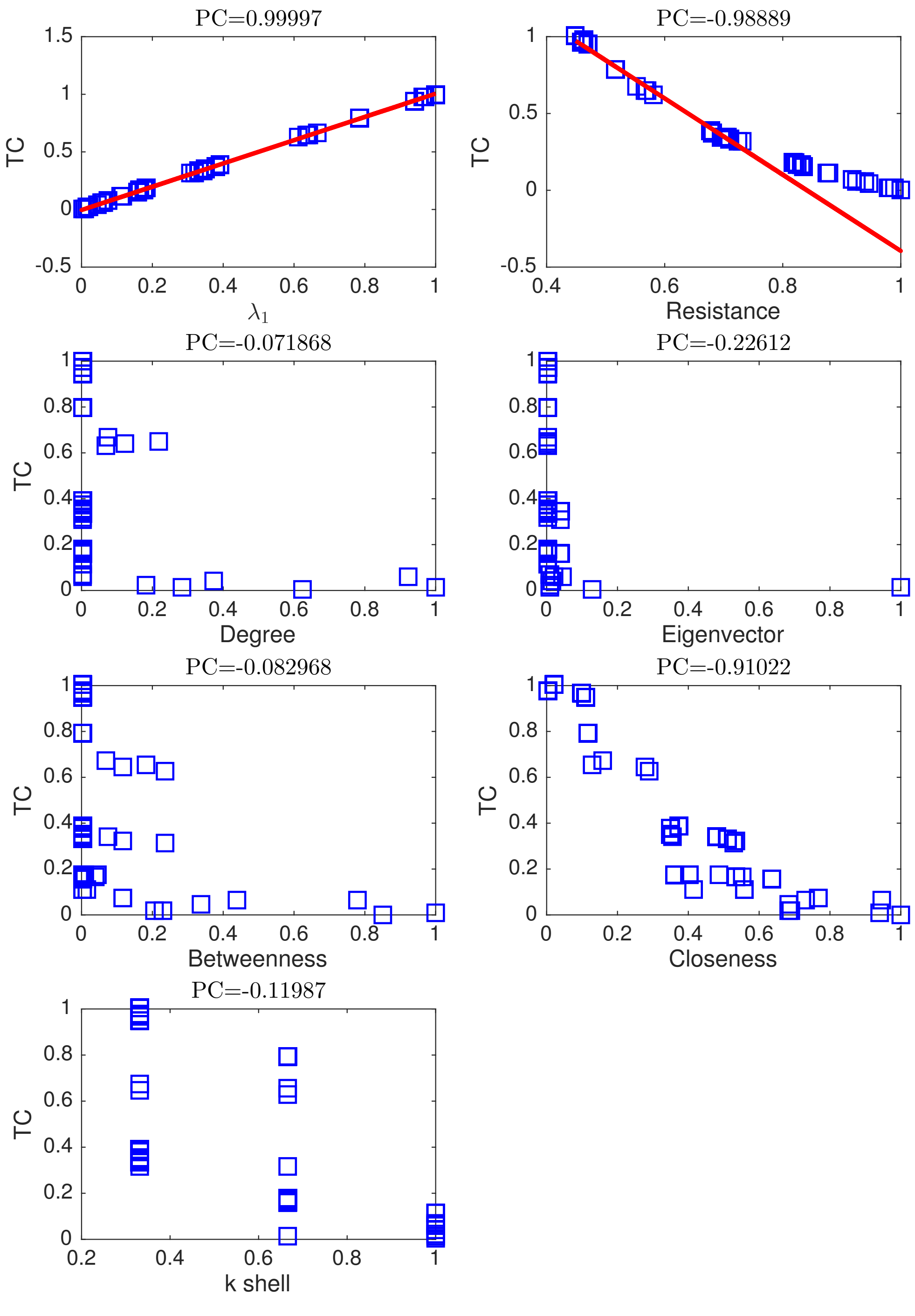

Figure 13: TC as a function of λ_1, resistance distance (RD), degree, eigenvector centrality, betweenness, closeness and k-shell with corresponding Pearson coefficient (PC) in Facebook network.

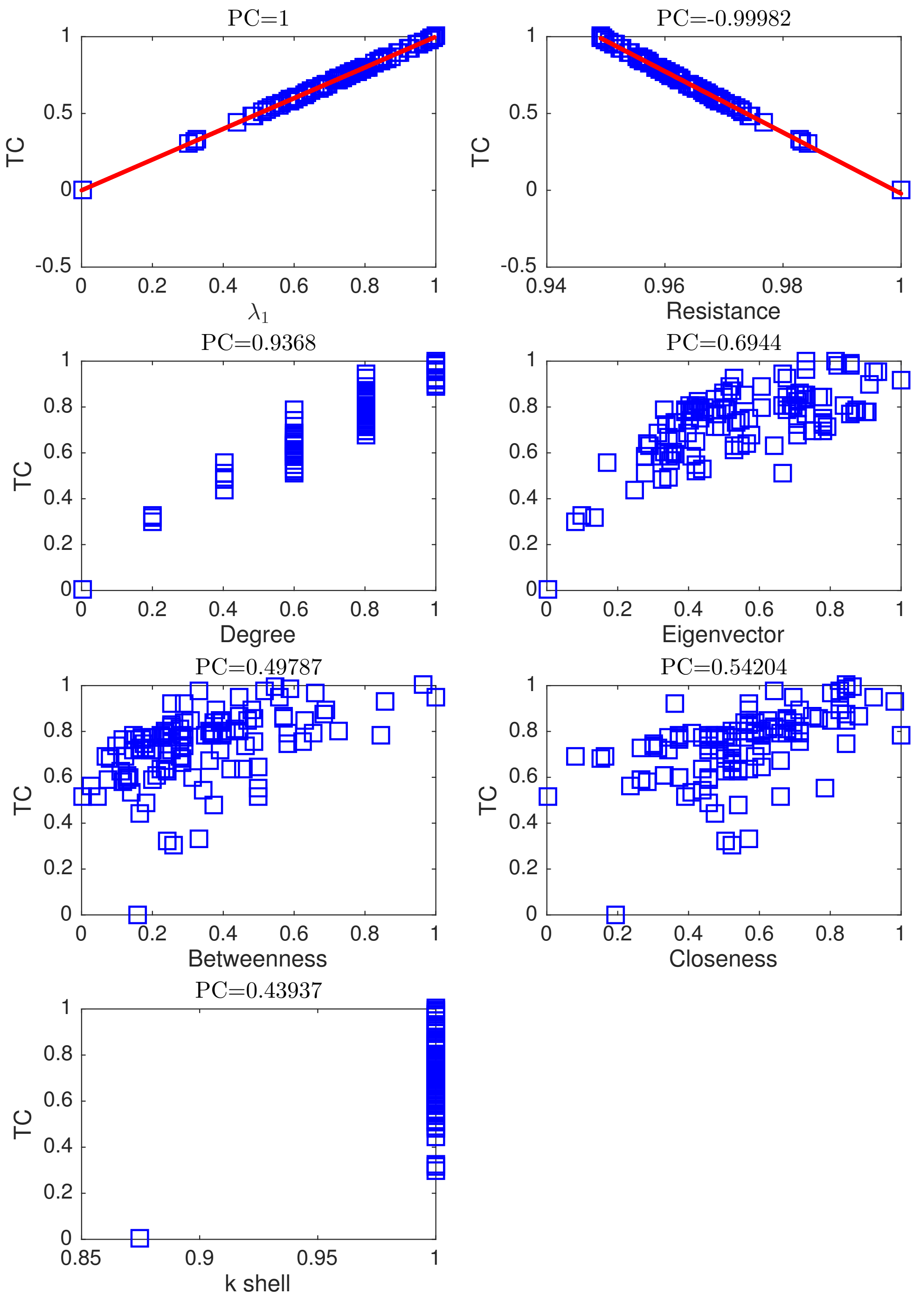

Figure 14: TC as a function of λ_1, resistance distance (RD), degree, eigenvector centrality, betweenness, closeness and k-shell with corresponding Pearson coefficient (PC) in Football network.

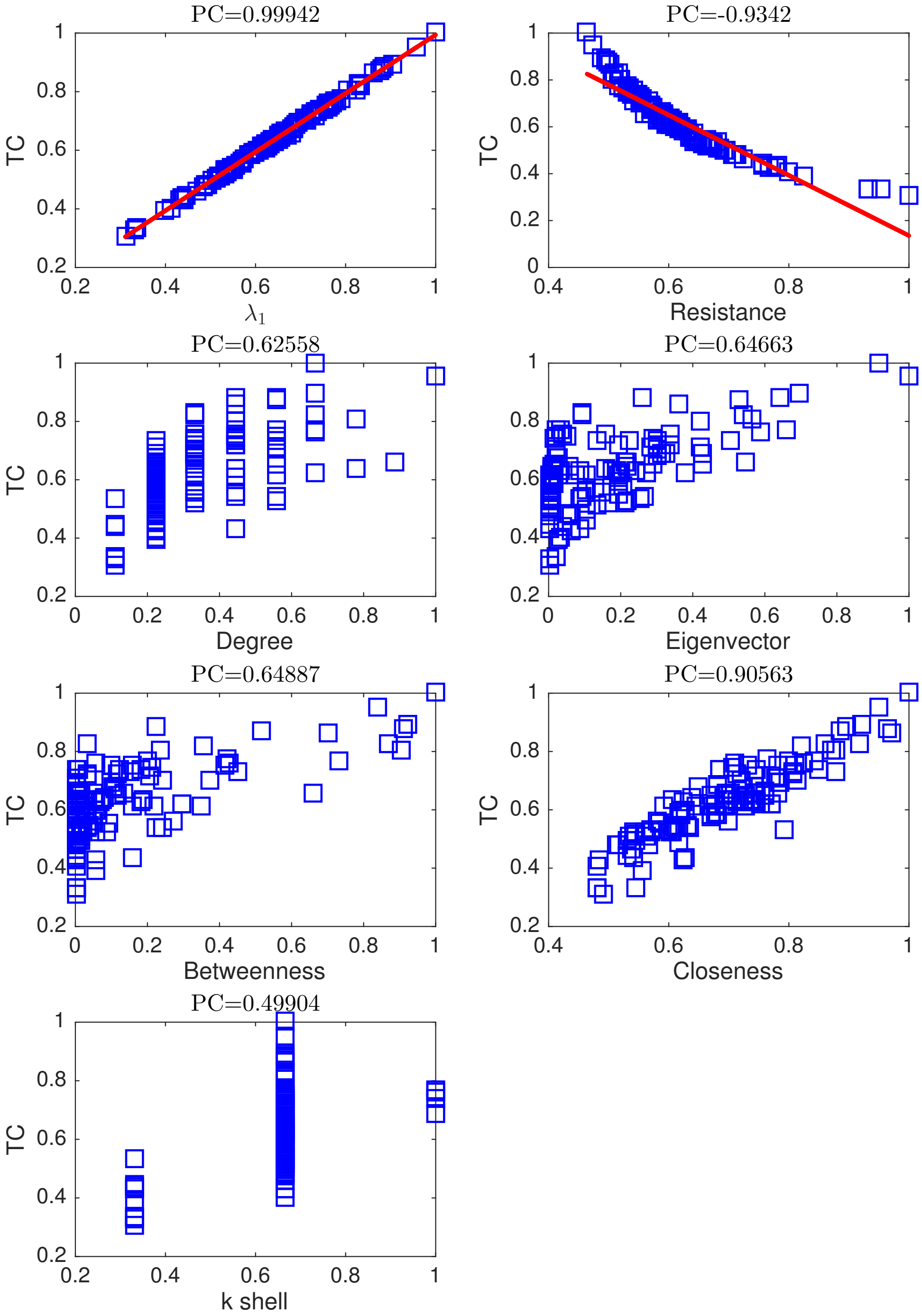

Figure 15: TC as a function of λ_1, resistance distance (RD), degree, eigenvector centrality, betweenness, closeness and k-shell with corresponding Pearson coefficient (PC) in IEEE 118 bus system network.

2.6 Correlation between ψ and m

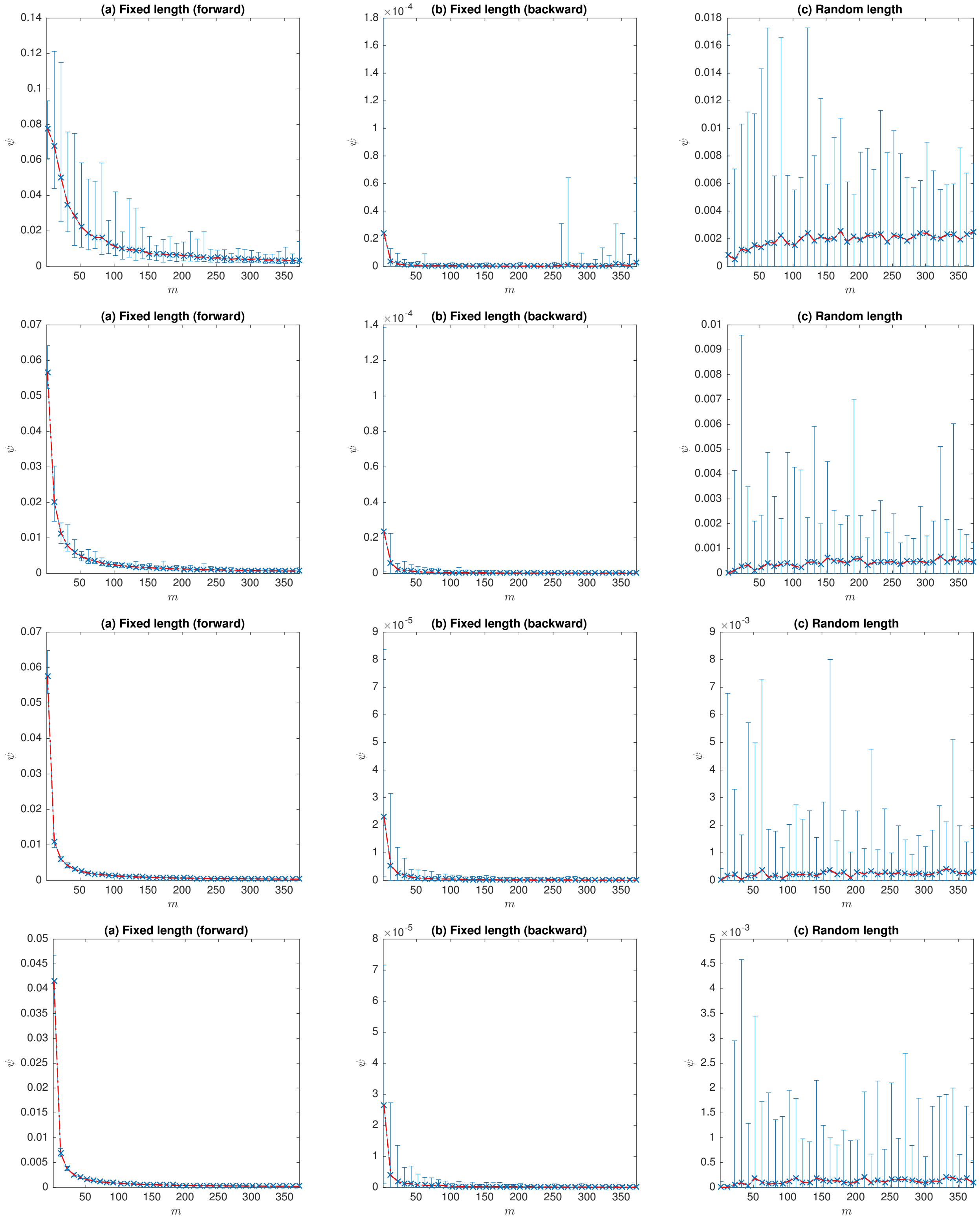

Figure 16: The error bar plot between the estimating error ψ and the length of snapshots m in 500-node ER random networks . The error bar is plotted with 500 realizations and one leader is randomly selected for each realization. The 4 rows are corresponding to $p = 0.1$, $p = 2.5$, $p = 0.5$ and $p = 0.75$, respectively. The 3 columns are corresponding to forward sampling, backward sampling and random sampling, respectively.

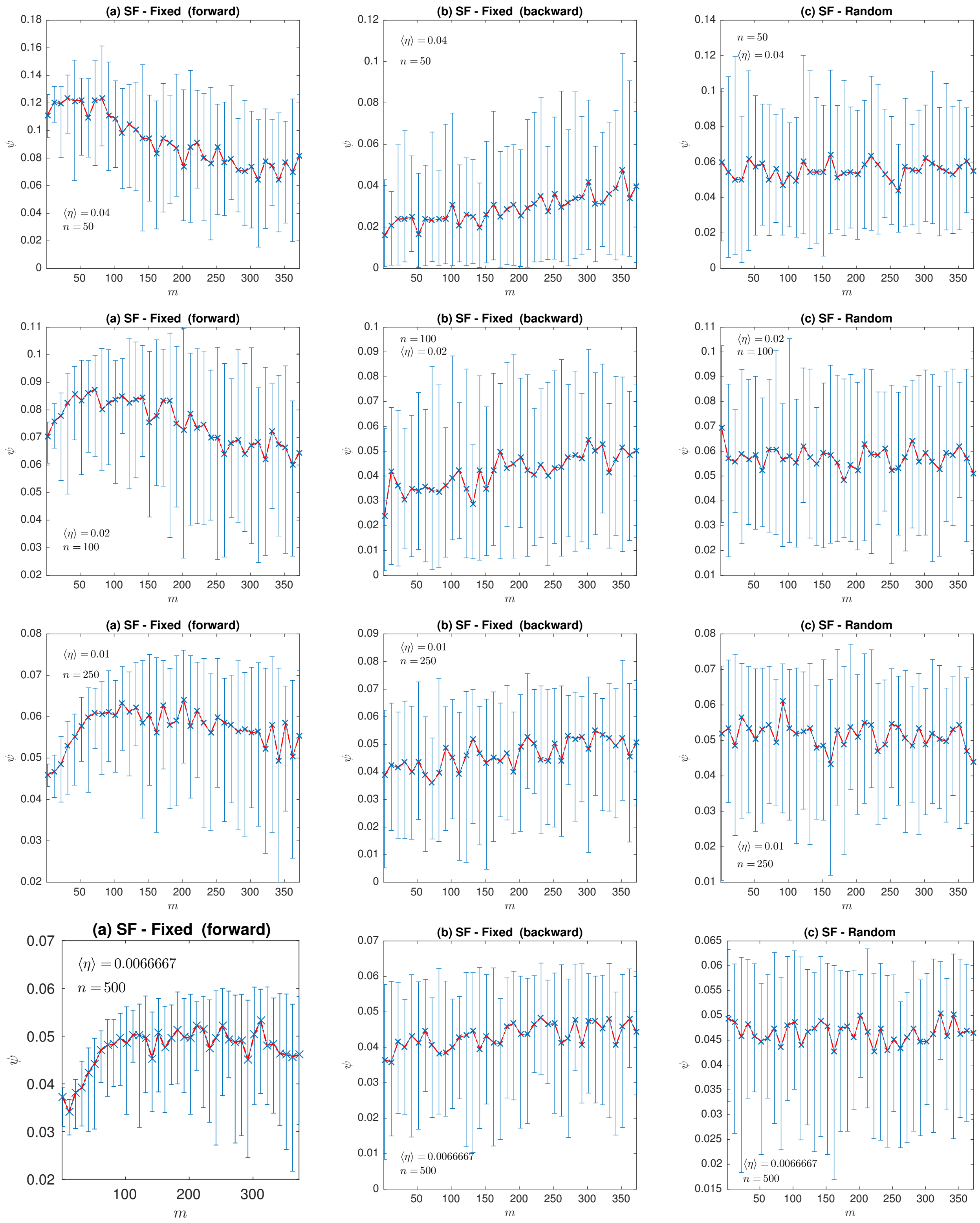

Figure 17: The error bar plot between the estimating error ψ and the length of snapshots m in scale-free networks with $n = 50$, $n = 100$, $n = 250$ and $n = 500$, respectively. The 3 columns are corresponding to forward sampling, backward sampling and random sampling, respectively.

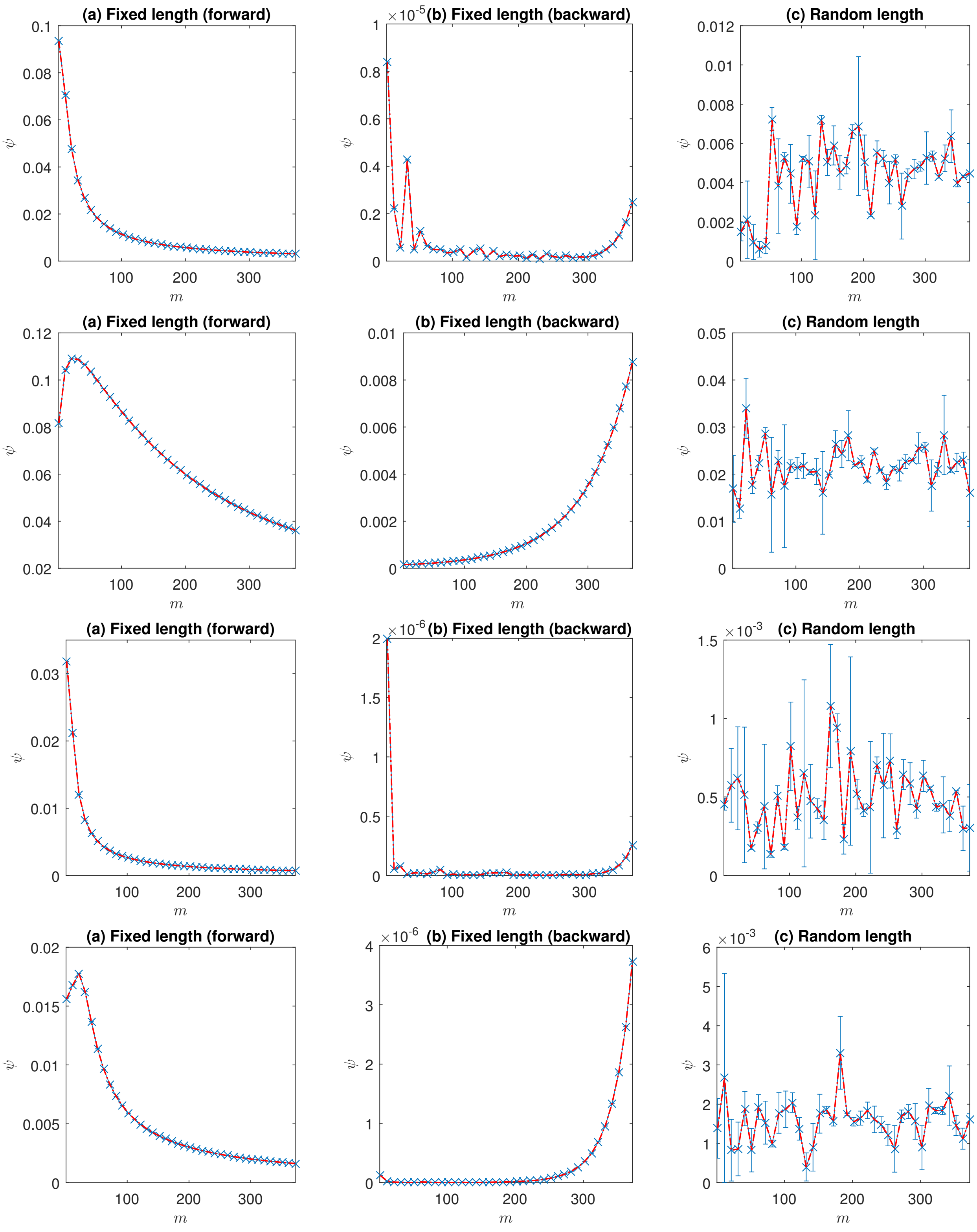

Figure 18: The error bar plot between the estimating error ψ and the length of snapshots m in 4 empirical networks. The 4 rows are corresponding to networks of Karate club, Contiguous USA, Jazz musicians and U. Rovira i Virgili, respectively. The 3 columns are corresponding to forward sampling, backward sampling and random sampling, respectively.

2.7 Approximation in sparse networks

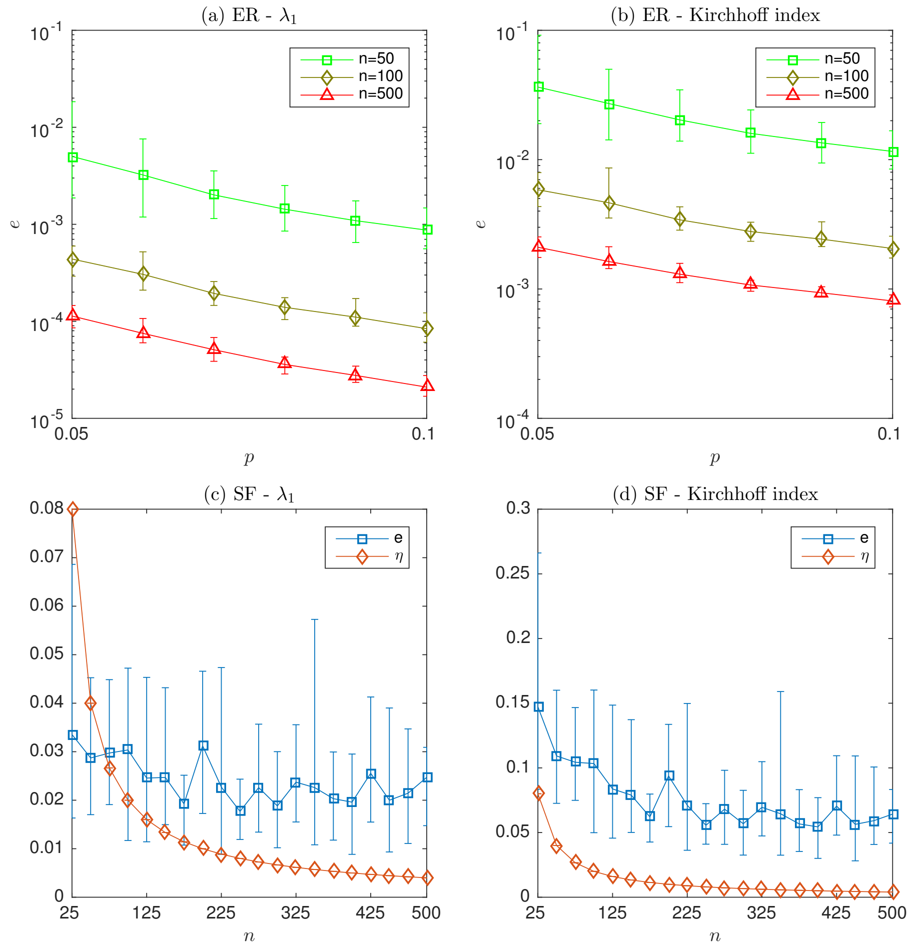

Figure 19: Error bar plot of the error of approximating λ_1 by $\frac{\alpha(\{i\})}{\sqrt{n}}$ in ER random networks (a) and scale-free networks (c). Error bar plot of the error of approximating Kirchhoff index by $\frac{1}{2}\left(\sum_{i=1}^{n}\frac{\sqrt{n}}{\alpha(\{i\})}-n^2\right)$ in ER random networks (b) and scale-free networks (d). The possibility for edge occurrence p ranges from 0.05 to 0.1 at the regular interval 0.01. For each realization of the network, we compute the TC for all nodes $i \in \mathcal{V}$ in the network and the corresponding $\lambda_1(\mathcal{L}_{\{i\}})$. Since the Kirchhoff index corresponds to an entire network, we only need to compute the Kirchhoff index for each realization of the network and its approximation by TC.

2.8 The distribution of entries in $\boldsymbol{v}_1(\mathcal{L}_{\mathcal{B}})$

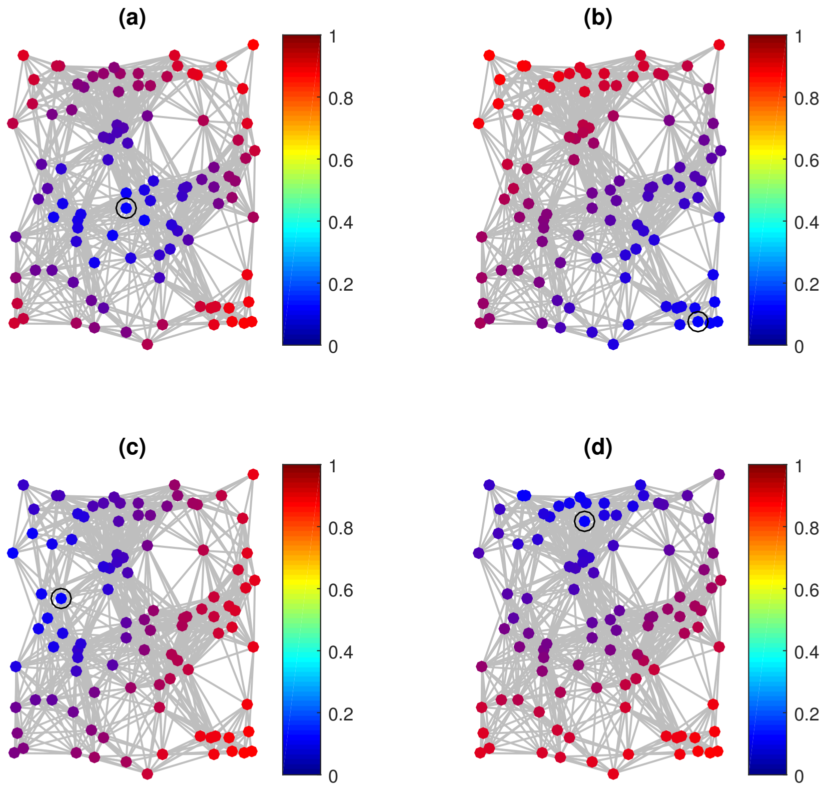

Figure 20: The distribution of entries in $\boldsymbol{v}_1(\mathcal{L}_{\mathcal{B}})$. (a),(b),(c),(d), External inputs influence the network via 4 different informed agents in the same network are shown. The informed agent is highlighted with the black circle in each panel. The colours of nodes in each panel range from the minimum to the maximum absolute value of entries in $\boldsymbol{v}_1$. The entry corresponding to the informed agent in $\boldsymbol{v}_1$ achieves the minimum in each panel. The location of spreader can therefore be identified.

2.9 Robustness

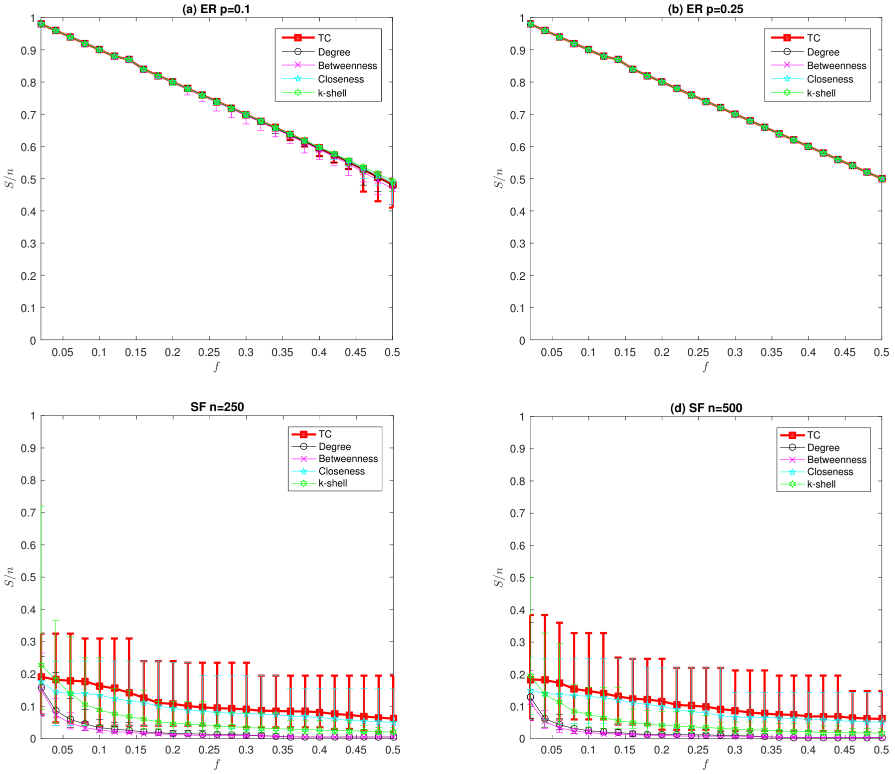

Figure 21: The effect of centrality attack $\frac{S}{n}$ as a function of the fraction of removed nodes f for centrality metrics including TC, degree, betweenness, closeness and k-shell. (a) the ER random network with $n = 500$ and $p = 0.1$, (b) the ER random network with $n = 500$ and $p = 0.25$, (c) the scale-free network with $n = 250$ and $\langle \eta \rangle = 0.008$, and (d) the scale-free network with $n = 500$ and $\langle \eta \rangle = 0.004$. The error bar plots are over 500 realizations for each network type.

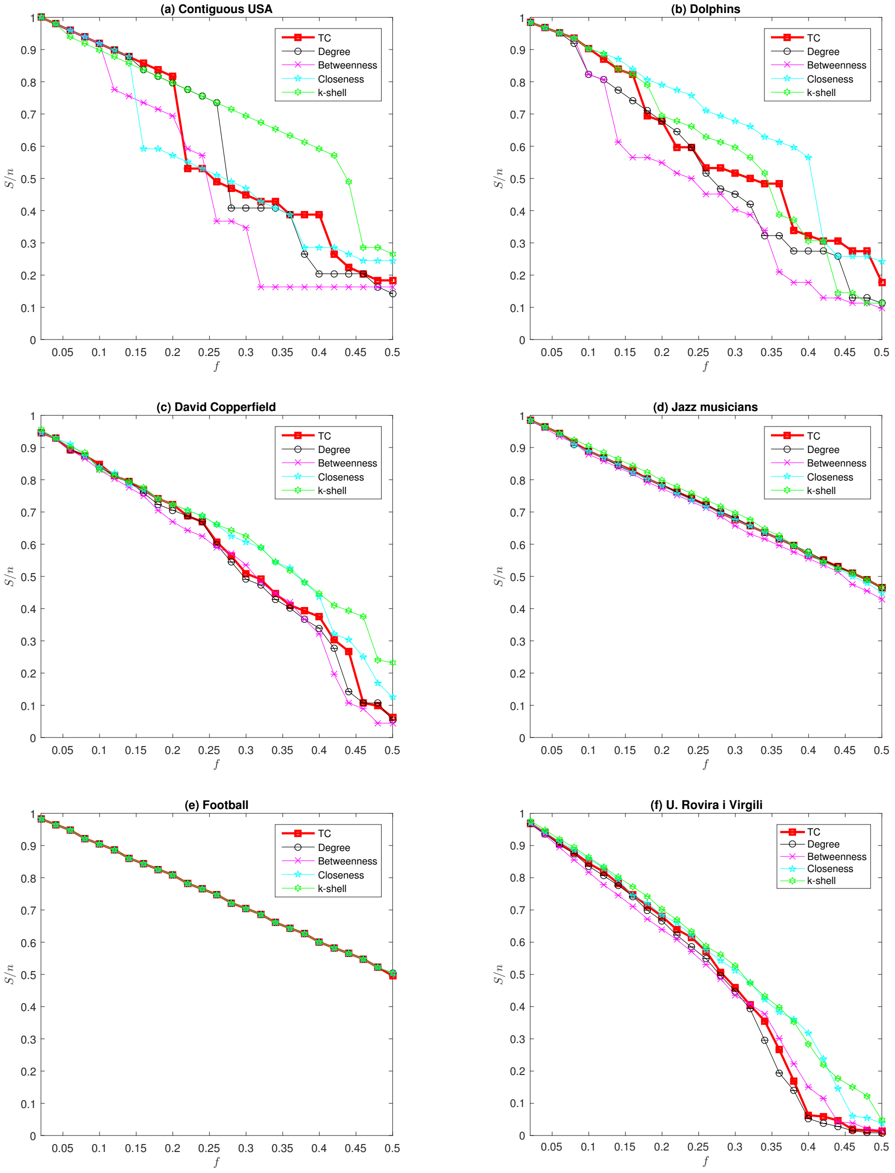

Figure 22: The effect of centrality attack $\frac{S}{n}$ as a function of the fraction of removed nodes f for centrality metrics including TC, degree, betweenness, closeness and k-shell in empirical networks. (a) the Contiguous USA network with $n = 49$, (b) the Dolphins network with $n = 62$, (c) the David Copperfield network with $n = 112$, (d) the Jazz musicians network with $n = 198$, (e) the American football network with $n = 115$, and (f) the U. Rovira i Virgili network with $n = 1133$.

Network				Pearson Coefficient						
				λ_1	RD	D	E	B	C	k
ER	$p = 0.05$,$n = 1000$,$\langle\eta\rangle = 0.05$			$1(2.65 \times 10^{-18})$	$-0.99(1.12 \times 10^{-4})$	$0.89(0.5 \times 10^{-3})$	$0.81(1.3 \times 10^{-3})$	$0.73(2.9 \times 10^{-3})$	$0.92(0.2 \times 10^{-3})$	$0.87(1.9 \times 10^{-3})$
	$p = 0.1$,$n = 1000$,$\langle\eta\rangle = 0.099$			$1(2.92 \times 10^{-19})$	$-0.99(3.92 \times 10^{-8})$	$0.92(1.1 \times 10^{-3})$	$0.88(1.2 \times 10^{-3})$	$0.80(2.8 \times 10^{-3})$	$0.92(0.3 \times 10^{-3})$	$0.82(8.4 \times 10^{-3})$
	$p = 0.25$,$n = 1000$,$\langle\eta\rangle = 0.25$			$1(1.74 \times 10^{-22})$	$-0.99(6.51 \times 10^{-12})$	$0.99(2.4 \times 10^{-5})$	$0.97(2.41 \times 10^{-3})$	$0.95(9.4 \times 10^{-5})$	$0.98(3.33 \times 10^{-3})$	$0.51(9.8 \times 10^{-3})$
	$p = 0.5$,$n = 1000$,$\langle\eta\rangle = 0.501$			$(1(6.09 \times 10^{-25})$	$-1(1.78(\times 10^{-14})$	$0.99(9.26 \times 10^{-7})$	$0.99(8.77 \times 10^{-7})$	$0.98(1.01 \times 10^{-5})$	$0.99(2.66 \times 10^{-6})$	NaN
SF	$n = 100$,$\langle\eta\rangle = 0.02$			$0.99(7.11 \times 10^{-7})$	$-0.92(9.55 \times 10^{-5})$	$0.54(3.64 \times 10^{-3})$	$0.82(1.56 \times 10^{-2})$	$0.61(3.14 \times 10^{-3})$	$0.99(2.49 \times 10^{-5})$	NaN
	$n = 250$,$\langle\eta\rangle = 0.008$			$0.99(1.18 \times 10^{-6})$	$-0.92(9.21 \times 10^{-5})$	$0.43(2.89 \times 10^{-3})$	$0.71(2.89 \times 10^{-2})$	$0.49(2.85 \times 10^{-3})$	$0.98(4.77 \times 10^{-5})$	NaN
	$n = 500$,$\langle\eta\rangle = 0.004$			$0.99(4.87 \times 10^{-7})$	$-0.93(8.99 \times 10^{-5})$	$0.37(1.3 \times 10^{-3})$	$0.7(8.3 \times 10^{-3})$	$0.41(1.07 \times 10^{-3})$	$0.98(1.52 \times 10^{-5})$	NaN
E	Dolphins	$n = 62$	$\eta = 0.084$	0.9999	-0.9780	0.8623	0.6956	0.5278	0.8738	0.9482
	U. Rovira i Virgili	$n = 1133$	$\eta = 0.0085$	1	-0.9716	0.7156	0.5724	0.4593	0.8453	0.9118
	Jazz musicians	$n = 198$	$\eta = 0.1406$	1	-0.9782	0.6033	0.5260	0.1813	0.7839	0.7507
	Karate club	$n = 34$	$\eta = 0.139$	0.9999	-0.9811	0.8380	0.9247	0.6806	0.8968	0.8998
	Contiguous USA	$n = 49$	$\eta = 0.091$	0.9999	-0.9479	0.7627	0.8659	0.5033	0.9359	0.8428
	US power grid	$n = 4941$	$\eta = 0.0005$	1	-0.9721	-0.4119	0.1466	-0.2346	-0.6902	-0.4682
	David Copperfield	$n = 112$	$\eta = 0.068$	1	-0.9774	0.6762	0.7071	0.4176	0.8409	0.9576
	Facebook (NIPS)	$n = 2888$	$\eta = 0.0007$	0.9999	-0.9888	-0.0719	-0.2261	-0.0830	-0.9102	-0.1199
	Football	$n = 115$	$\eta = 0.0935$	1	-0.9998	0.9368	0.6944	0.4979	0.5420	0.4394
	IEEE 118 Bus	$n = 118$	$\eta = 0.0259$	0.9994	-0.9342	0.6256	0.6466	0.6489	0.9056	0.4990

Table 1: The Pearson coefficient between TC and λ_1, RD, degree (D), eigenvector (E), betweenness (B), closeness (C) and k-shell (k) in ER random networks (ER), scale-free networks (SF) and empirical networks (E). The digitals in the table represent Pearson coefficient and regression coefficient of corresponding cases (for instance, 2.14×10^{-14} represents the mean of the data is 1 with a variance of 2.14×10^{-14}). For ER random networks and scale-free networks, the mean and variance are across 500 realizations for each network type.

3 Supplementary Tables

3.1 Pearson Correlation Analysis

Network				Regression Coefficient λ_1	Regression Coefficient RD
ER	$p = 0.05$,$n = 1000$,$\langle\eta\rangle = 0.05$			$1(1.2007 \times 10^{-9})$	$-1.17(1.45 \times 10^{-3})$
ER	$p = 0.1$,$n = 1000$,$\langle\eta\rangle = 0.099$			$1.0003(2.0075 \times 10^{-10})$	$-1.0709(1.14 \times 10^{-4})$
ER	$p = 0.25$,$n = 1000$,$\langle\eta\rangle = 0.25$			$1(3.89 \times 10^{-12})$	$-1(1.79 \times 10^{-6})$
ER	$p = 0.5$,$n = 1000$,$\langle\eta\rangle = 0.501$			$1(2.60 \times 10^{-13})$	$-1.01(1.14 \times 10^{-7})$
SF	$n = 100$,$\langle\eta\rangle = 0.02$			$0.99(2.52 \times 10^{-4})$	$-1.07(4.08 \times 10^{-3})$
SF	$n = 250$,$\langle\eta\rangle = 0.008$			$0.99(3.43 \times 10^{-4})$	$-1.02(5.36 \times 10^{-3})$
SF	$n = 500$,$\langle\eta\rangle = 0.004$			$1(3.12 \times 10^{-4})$	$-0.99(5.12 \times 10^{-3})$
E	Dolphins	$n = 62$	$\eta = 0.084$	1.0004	-2.2955
E	U. Rovira i Virgili	$n = 1133$	$\eta = 0.0085$	1	-2.2224
E	Jazz musicians	$n = 198$	$\eta = 0.1406$	1.0001	-2.1591
E	Karate club	$n = 34$	$\eta = 0.139$	1.0019	-2.8238
E	Contiguous USA	$n = 49$	$\eta = 0.091$	1.0082	-2.0916
E	US power grid	$n = 4941$	$\eta = 0.0005$	0.9985	-1.0672
E	David Copperfield	$n = 112$	$\eta = 0.068$	1.0001	-2.1396
E	Facebook (NIPS)	$n = 2888$	$\eta = 0.0007$	1.0101	-2.483
E	Football	$n = 115$	$\eta = 0.0935$	1.0001	-19.8944
E	IEEE 118 Bus	$n = 118$	$\eta = 0.0259$	0.9999	-1.2871

Table 2: The regression coefficient between TC and λ_1 and RD in ER random networks (ER), scale-free networks (SF) and empirical networks (E). The digitals in the table represent the Pearson coefficient and the regression coefficient for corresponding cases (for instance, $1(1.80 \times 10^{-8})$ represents the mean of the data is 1 with a variance of 1.80×10^{-8}).

3.2 Approximation in empirical networks

**Network**	n	η	λ_1	K
Dolphins	62	0.084	0.29%	2.67%
U. Rovira i Virgili	1,133	0.0085	0.004%	0.05%
Jazz musicians	198	0.1406	0.01%	0.25%
Zachary's karate club	34	0.139	0.21%	2.31%
Contiguous USA	49	0.091	0.84%	7.23%
David Copperfield	112	0.068	0.03%	0.49%
Facebook (NIPS)	2,888	0.0007	0.20%	1.19%
Football	115	0.0935	0.01%	0.22%
IEEE 118 bus system	118	0.0259	1.51%	7.57%

Table 3: The relative error of approximating λ_1 and Kirchhoff index (K) with TC for 9 empirical networks.
